# Supplementary material for: Machine intelligence accelerated design of conductive MXene aerogels with programmable properties
Source: Nat Commun. 2024 Jun 1;15:4685. doi: 10.1038/s41467-024-49011-8 (PMC11144242; doi:10.1038/s41467-024-49011-8)
Supplement: Supplementary file 1 — Supplementary Information [file 41467_2024_49011_MOESM1_ESM.docx]

SUPPORTING INFORMATION

**Machine Intelligence Accelerated Design of Conductive
MXene Aerogels with Programmable Properties**

Snehi Shrestha^1^, Kieran James Barvenik^2^, Tianle Chen^1^, Haochen Yang^1^, Yang Li^1^, Meera Muthachi Kesavan^1^, Joshua M. Little^1^, Hayden C. Whitley^1^, Zi Teng^3^, Yaguang Luo^3^, Eleonora Tubaldi^2,4,*^, Po-Yen Chen^1,4,*^

^1^ Department of Chemical and Biomolecular Engineering, University of Maryland, College Park, MD 20742, United States

^2^ Department of Mechanical Engineering, University of Maryland, College Park, MD 20742, United States

^3^ Food Quality Laboratory and Environment Microbial Food Safety Laboratory, US Department of Agriculture, Agricultural Research Service, Beltsville Agricultural Research Center, Beltsville, MD 20725, United States

^4^ Maryland Robotics Center, College Park, MD 20742, United States

* Email: [etubaldi@umd.edu](mailto:etubaldi@umd.edu) (E.T.)

[checp@umd.edu](mailto:checp@umd.edu) (P.-Y. C.)

| Supplementary Fig. 1 | Robot–human teaming workflow for producing conductive aerogels. | 4 | |  |
| --- | --- | --- | --- | --- |
| Supplementary Fig. 2 | Schematic illustration of a model expansion strategy. | 4 | |  |
| Supplementary Fig. 3 | TEM images of MXene nanosheets and cellulose nanofibers (CNFs). | 5 | |  |
| Supplementary Fig. 4 | Fabrication of conductive aerogels with tunable compositions and mixture loadings. | 6 | |  |
| Supplementary Fig. 5 | Tunable mechanical and electrical properties of conductive aerogels. | 7 | |  |
| Supplementary Fig. 6 | Relationship between step size and number of data points required for an extensive dataset. | 9 | |  |
| Supplementary Fig. 7 | Comparison of prediction models using different mechanical labels. | 10 | |  |
| Supplementary Fig. 8 | Feasible parameter space of conductive aerogels. | 11 | |  |
| Supplementary Fig. 9 | Data distribution plots with MXene and gelatin loadings as the axes. | 12 | |  |
| Supplementary Fig. 10 | Ti 2*p* spectra of MXene/CNF aerogels without and with GA incorporation. | 13 | |  |
| Supplementary Fig. 11 | Empirical evidence for implementing the UIP Method. | 14 | |  |
| Supplementary Fig. 12 | 2D Voronoi tessellation diagrams from loop #1 to loop #8. | 15 | |  |
| Supplementary Fig. 13 | Distribution profiles of data points collected from different sampling methods. | 16 | |  |
| Supplementary Fig. 14 | Prediction model performance based on different sampling methods. | 17 | |  |
| Supplementary Fig. 15 | SEM images of model-suggested conductive aerogels. | 18 | |  |
| Supplementary Fig. 16 | Working mechanism of Shapley Additive exPlanations (SHAP). | 19 | |  |
| Supplementary Fig. 17 | SHAP values of MXene, CNF, gelatin, and GA loadings and the mixture loadings on $R_{0}$ values of conductive aerogels. | 20 | |  |
| Supplementary Fig. 18 | Positive relationships between aerogel density and mixture loading. | 21 | |  |
| Supplementary Fig. 19 | Dependence of electrical resistances and aerogel microstructures on mixture loading. | 22 | |  |
| Supplementary Fig. 20 | Numerical geometries for FE models. | 23 | |  |
| Supplementary Fig. 21 | High-, medium-, low-density aerogel models at their relaxed states. | 24 | |  |
| Supplementary Fig. 22 | Joule’s law of strain-insensitive conductive aerogels. | 25 | |  |
| Supplementary Fig. 23 | Comparison of strain-insensitive conductive aerogels with commercial foam materials in terms of thermal conductivity and density. | 26 | |  |
|  |  |  | |  |
|  |  |  | |  |
| Supplementary Note 1 | Rationale of building block selection and model expansion strategy. | 27 | |  |
| Supplementary Note 2 | Estimated number of experiments required to build an extensive dataset for conductive aerogels. | 29 | |  |
| Supplementary Note 3 | Multi-stage AI/ML framework. | 30 | |  |
| Supplementary Note 4 | Classification standards used for categorizing 264 samples. | 32 | |  |
| Supplementary Note 5 | Training of a SVM classifier. | 33 | |  |
| Supplementary Note 6 | Implementation of UIP method. | 34 | |  |
| Supplementary Note 7 | Calculation of A Score acquisition function. | 35 | |  |
| Supplementary Note 8 | Shapley Additive exPlanations (SHAP) model interpretation method. | 37 | |  |
| Supplementary Note 9 | FE model construction for conductive aerogels. | 39 | |  |
|  |  |  | |  |
|  |  |  | |  |
| Supplementary Table 1 | Description of independent and dependent variables. | 42 | |  |
| Supplementary Table 2 | Discrete grades of 264 conductive aerogels with different MXene/CNF/gelatin ratios. | 43 | |  |
| Supplementary Table 3 | Testing data points for the SVM classifier. | 50 | |  |
| Supplementary Table 4 | Training data points for the prediction model. | 52 | |  |
| Supplementary Table 5 | Testing data points for the prediction model. | 57 | |  |
| Supplementary Table 6 | Training dataset for various prediction models based on different sampling methods. | 58 | |  |
| Supplementary Table 7 | Comparison between experimental and model-predicted $\sigma_{30}$ and $R_{0}$ values in Fig. 3a. | 60 | |  |
| Supplementary Table 8 | Property requirements, model-suggested fabrication parameters, and experimental results in Fig. 3c. | 61 | |  |
| Supplementary Table 9 | Structural features of conductive aerogels extracted from the SEM images in Fig. 4c. | 62 | |  |
| Supplementary Table 10 | Compositions and mixture loadings of conductive aerogels suggested by the champion model for wearable heating in Fig 5b. | 63 | |  |
| Supplementary Table 11 | Comparison of our AI/ML framework with the state-of-the-art works. | 64 | |  |
| Supplementary Table 12 | Comparison of our robotics/ML-integrated workflow with the state-of-the-art works regarding the production of conductive MXene aerogels. | 65 | |  |
| Supplementary Table13 | Comparison of our AI/ML and data-driven approach with the state-of-the-art works regarding design insight elucidation. | 68 | |  |
|  |  |  | |  |
|  |  |  | |  |
| Supplementary Movie 1 | Automatic pipetting robot (i.e., OT-2 robot) capable of preparing mixed dispersions with various MXene/CNF/gelatin/GA ratios and mixture loadings. | 71 | |  |
| Supplementary Movie 2 | UR5e robotic arm capable of automating the compression tests of conductive MXene aerogels. | 71 | |  |
|  |  |  | |  |
|  |  |  | |  |
| Supporting References | | | 72 | |


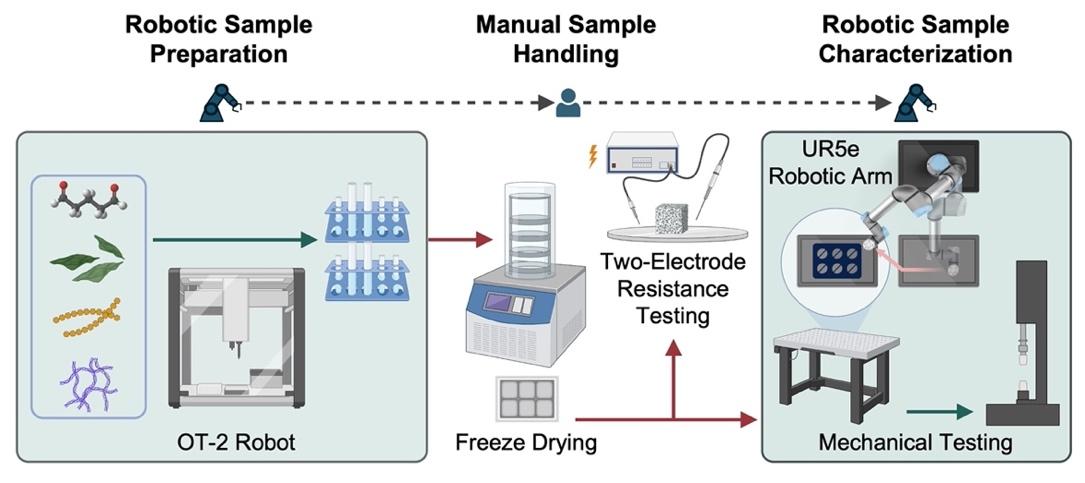


**Supplementary Fig. 1. Robot–human teaming workflow for producing conductive aerogels.** There are no robotic platforms that fully automate the entire freeze-drying process. We have automated the preparation of aqueous mixtures and the execution of compression tests through the use of an OT-2 robot and a UR5e-automated compression tester, respectively.

**
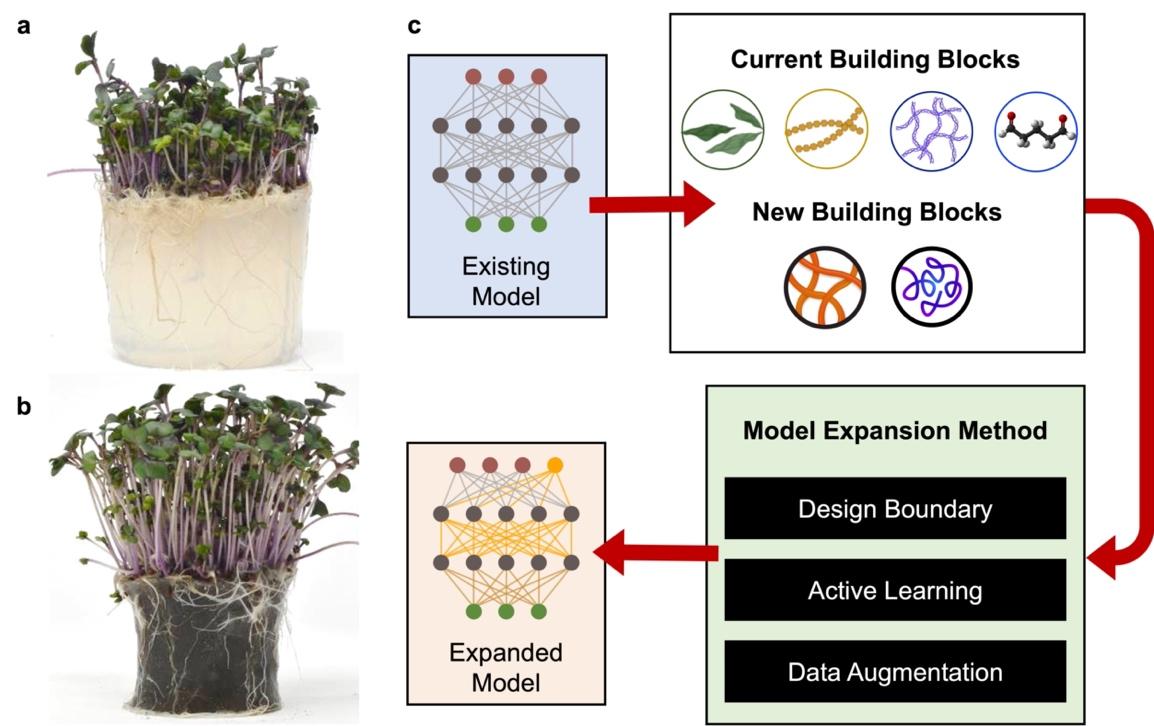
**

**Supplementary Fig. 2. Schematic illustration of a model expansion strategy.** Photos of microgreens grown on hydrogel media (**a**) without and (**b**) without the nanofillers. (**c**) Workflow of a model expansion method.


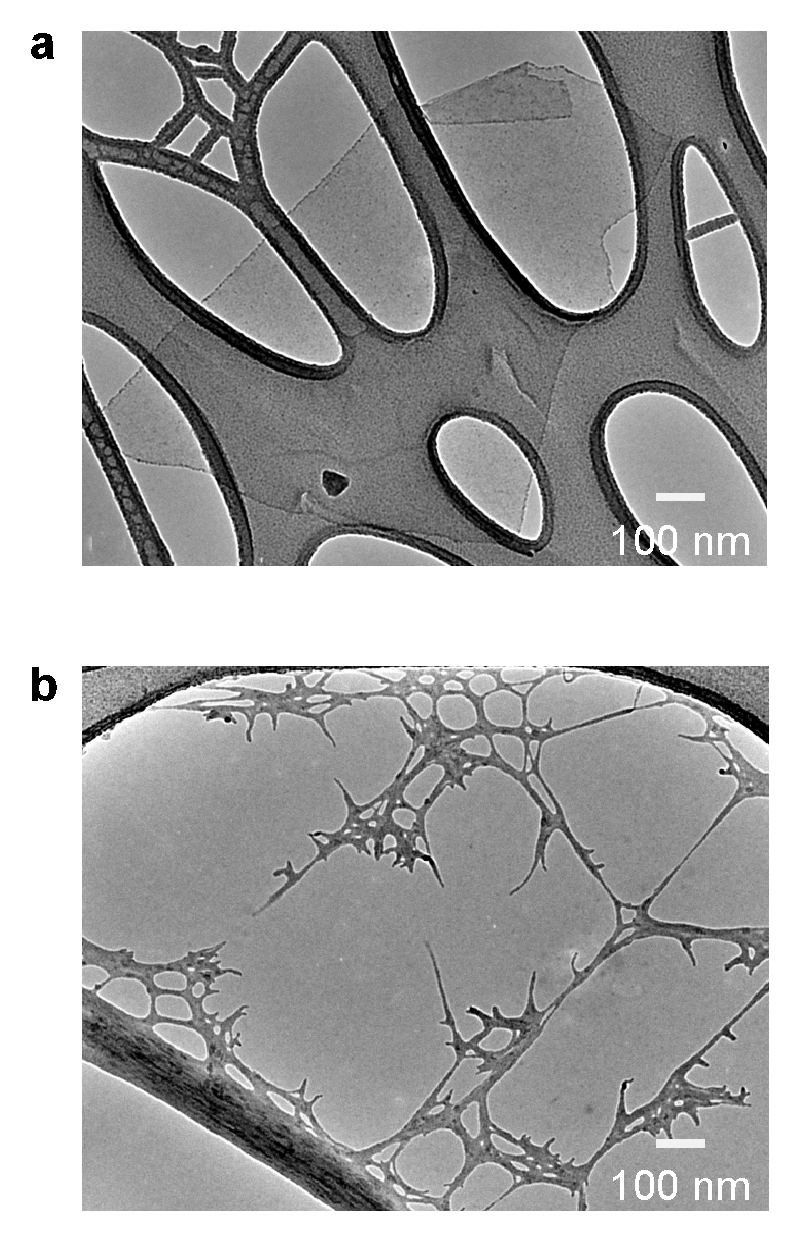


**Supplementary Fig. 3. TEM images of MXene nanosheets and cellulose nanofibers (CNFs).** (**a**) TEM image of MXene nanosheets. (**b**) TEM image of CNFs.

**
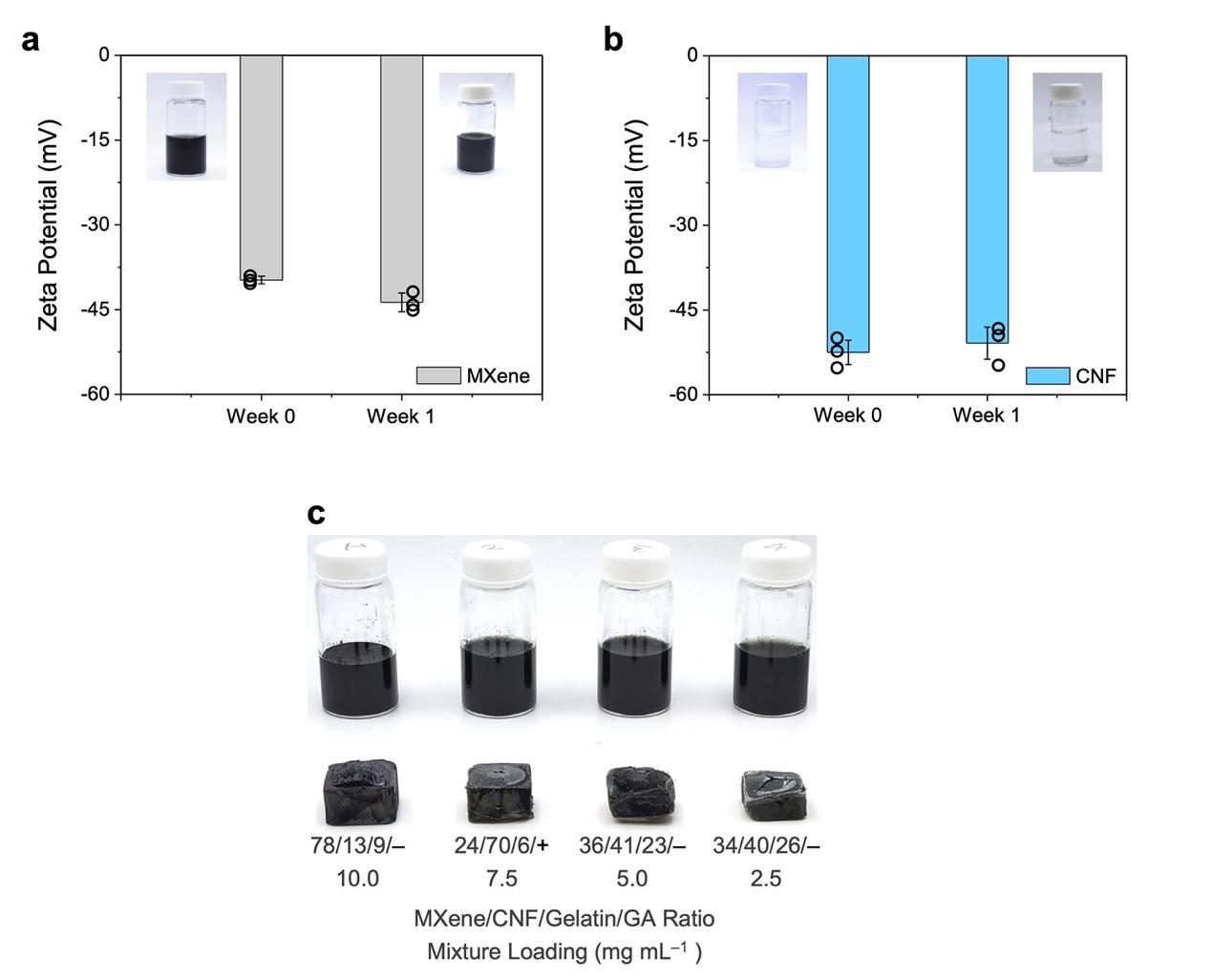
**

**Supplementary Fig. 4. Fabrication of conductive aerogels with tunable compositions and mixture loadings.** (**a**) Zeta potentials of MXene before and after one-week storage. Data are presented as mean ± s.d., *n* = 3, with each independent experiment marked by an open black circle. (**b**) Zeta potentials of CNF dispersions before and after one-week storage. Data are presented as mean ± s.d., *n* = 3, with each independent experiment marked by an open black circle. (**c**) Photo of various MXene/CNF/gelatin/ glutaraldehyde (GA) mixtures with various ratios and mixture loadings as well as the resulting conductive aerogels. Error bars represent s.d.


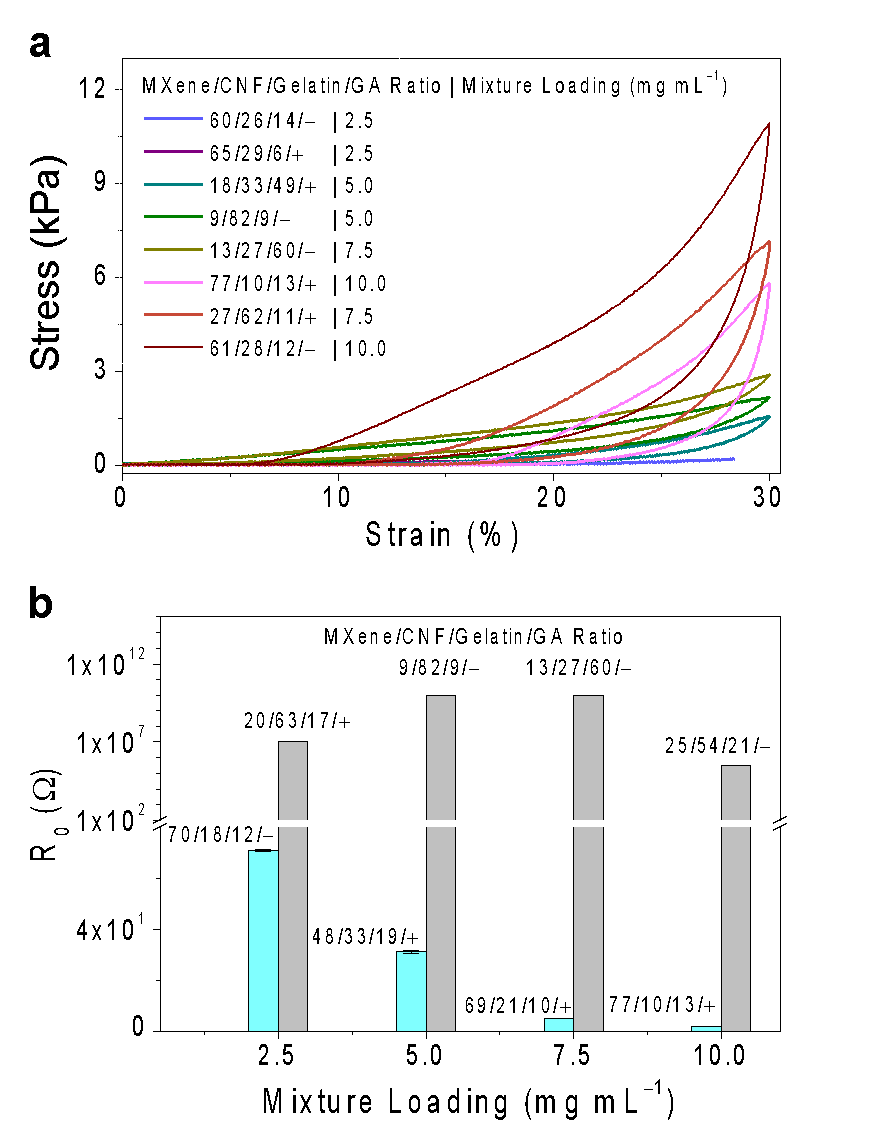


**Supplementary Fig. 5. Tunable mechanical and electrical properties of conductive aerogels.** The mechanical and electrical properties of conductive aerogels, such as $\sigma_{30}$ and $R_{0}$, showed complex and nonlinear correlations with the MXene/CNF/gelatin/GA ratios and the solid contents of aqueous mixtures (abbreviated as mixture loadings). (**a**) Stress–strain curves of eight conductive aerogels with different MXene/CNF/gelatin/GA ratios and mixture loadings. (**b**) $R_{0}$ values of eight conductive MXene aerogels with different MXene/CNF/gelatin/GA ratios and mixture loadings. It is worth mentioning that, with regard to the fabrication processes of conductive aerogels, both freeze drying temperature and pressure were held constant at –80 °C and 0.3 Pa, respectively. Therefore, they were not considered as independent variables.


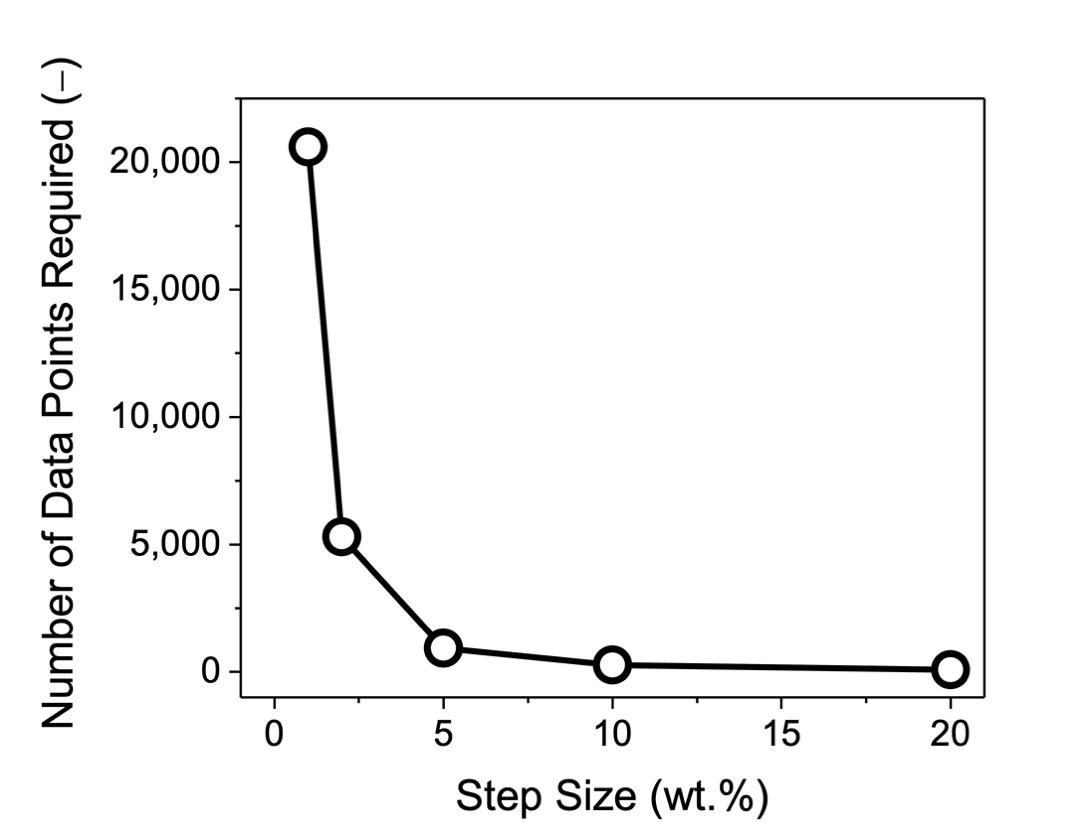


**Supplementary Fig. 6. Relationship between step size and number of data points required for an extensive dataset.**


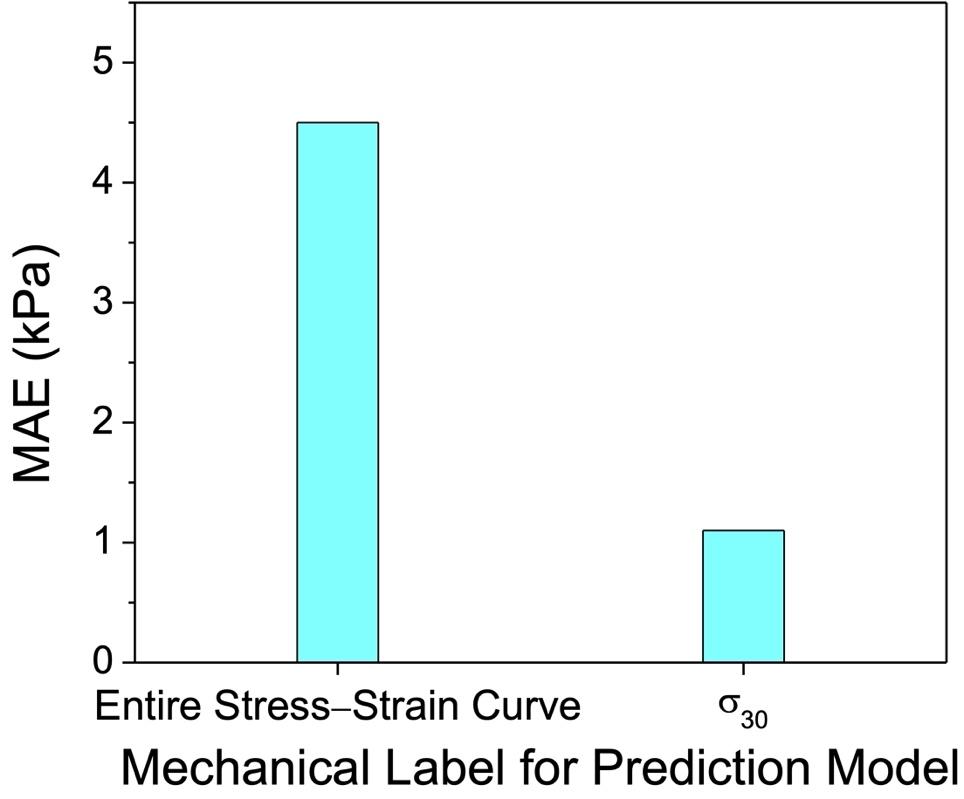


**Supplementary Fig. 7. Comparison of prediction models using different mechanical labels.** When the entire stress-strain curve was used as the mechanical labels for training data points, the prediction model demonstrated a higher MAE value of 4.5 kPa. On the other hand, when the
$\sigma_{30}$ value was used as the mechanical labels for training data points, the prediction model demonstrated a lower MAE value of 1.1 kPa.

**
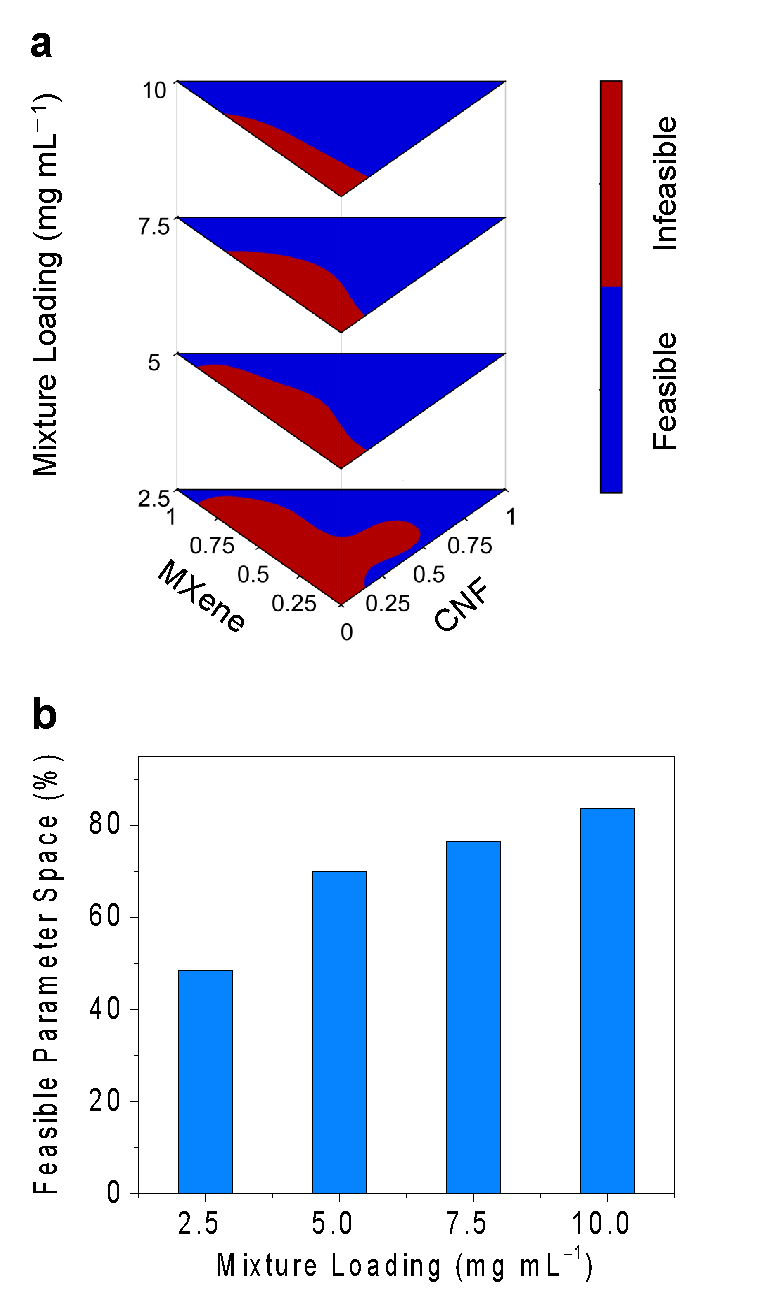
**

**Supplementary Fig. 8. Feasible parameter space of conductive aerogels.** (**a**) Binary maps representing the feasible parameter space of obtaining A-grade conductive aerogels. (**b**) As the mixture loading decreased from 10.0 to 2.5 mg mL^–1^, the area of the feasible parameter space decreased from 83.7% to 48.6%, respectively. It is worth mentioning that, with regard to the fabrication processes of conductive aerogels, both freeze drying temperature and pressure were held constant at –80 °C and 0.3 Pa, respectively. Therefore, they were not considered as independent variables.


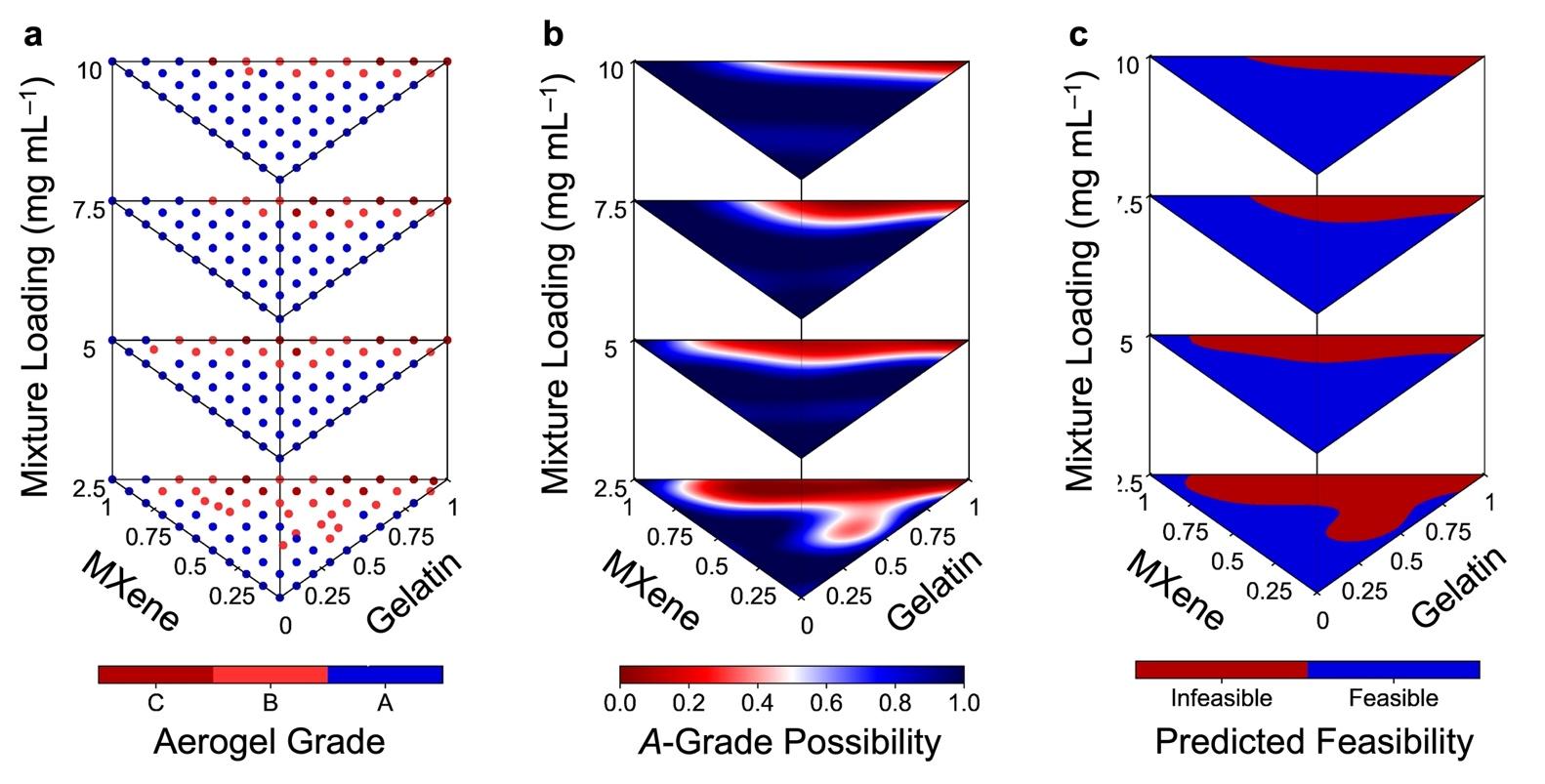


**Supplementary Fig. 9. Data distribution plots with MXene and gelatin loadings as the axes.** (**a**) 264 MXene/CNF/gelatin aerogels with different grades based on their structural integrity and monolithic nature. (**b**) Four heatmaps showcasing the possibilities of producing A-grade conductive aerogels at specific MXene/CNF/gelatin ratios and mixture loadings. (**c**) Binary maps representing the feasible parameter space of obtaining A-grade conductive aerogels.

**
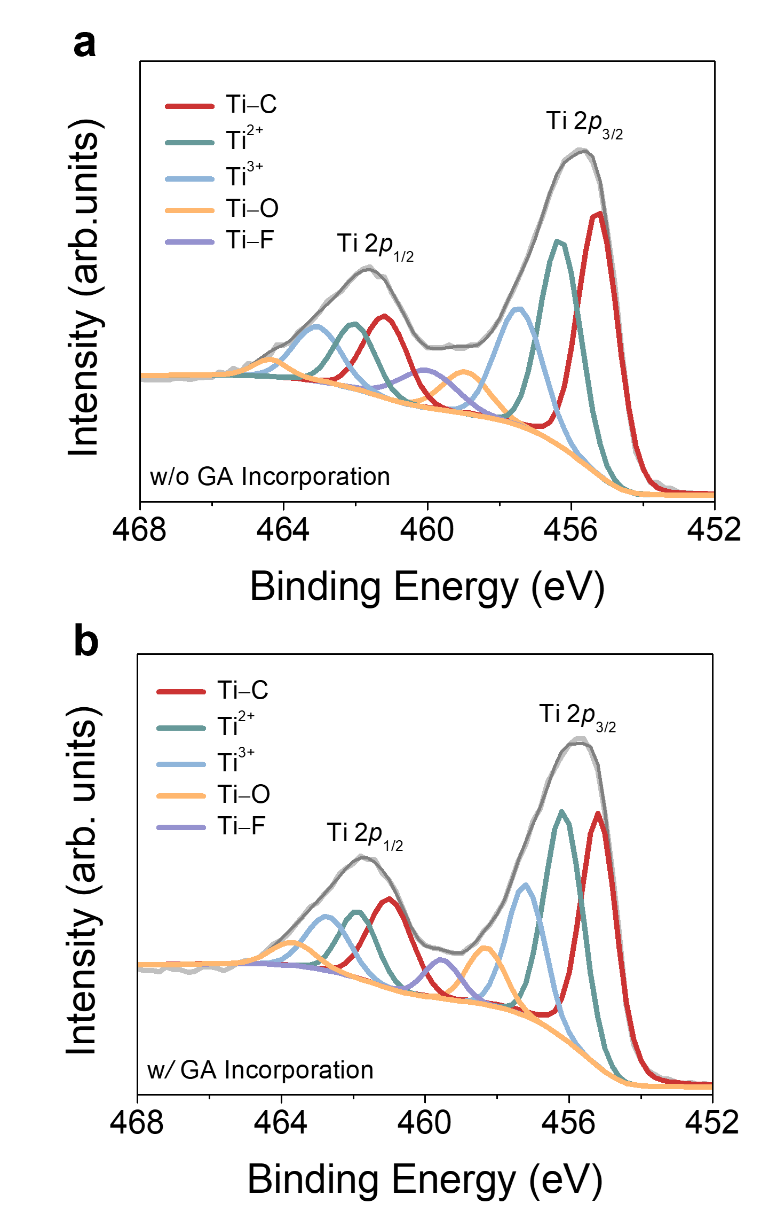
**

**Supplementary Fig. 10. Ti 2*p* spectra of MXene/CNF aerogels without and with GA incorporation.** (**a**) Ti 2*p* spectrum of the conductive aerogel without GA incorporation (at the MXene/CNF/gelatin/GA ratio of 80/20/0/– and the mixture loading of 10 mg mL^–1^). (**b**) Ti 2*p* spectrum of the conductive aerogel with GA incorporation (at the MXene/CNF/gelatin/GA ratio of 80/20/0/+ and the mixture loading of 10 mg mL^–1^).

**
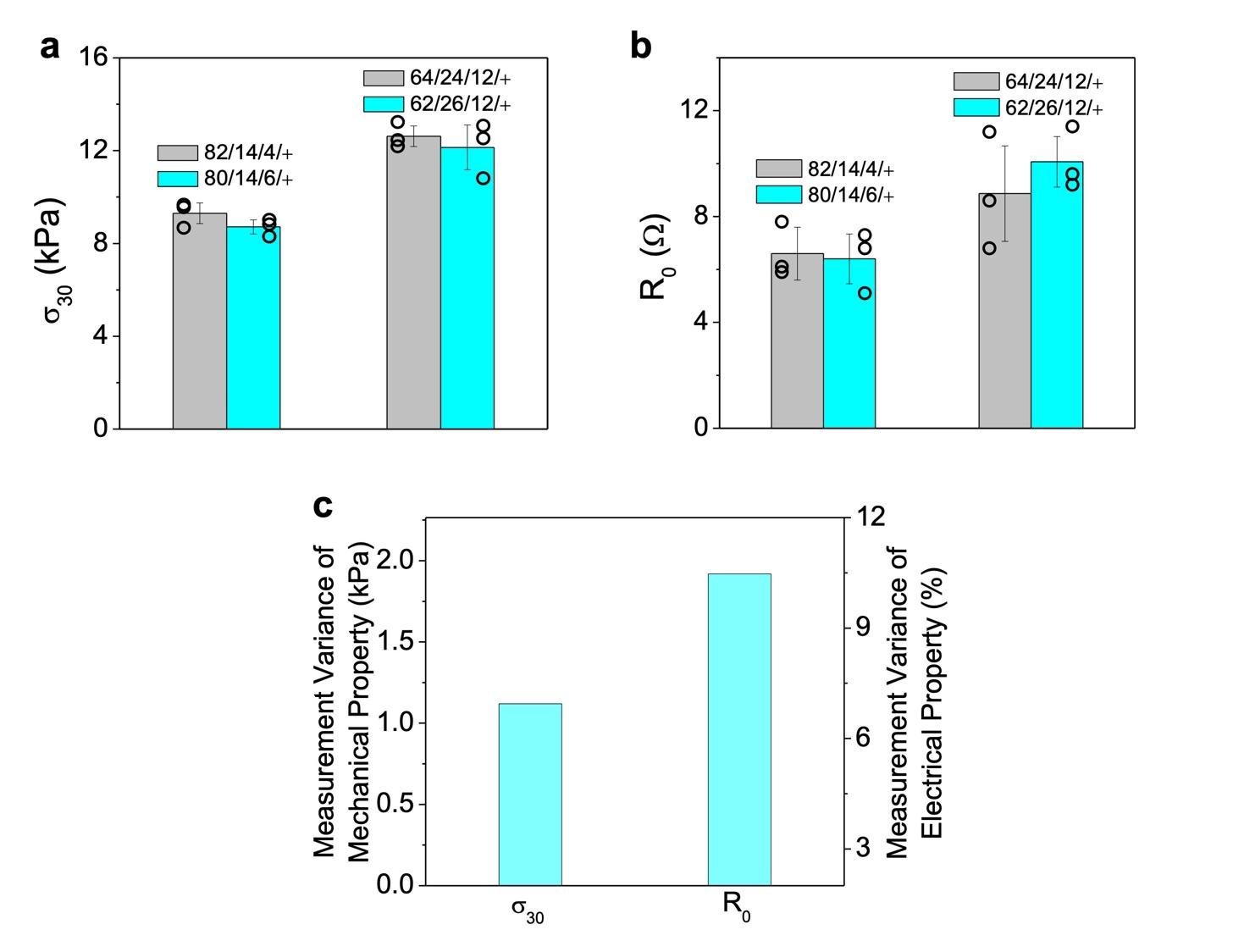
**

**Supplementary Fig. 11. Empirical evidence for implementing the UIP Method.** With slight variations of MXene/CNF/gelatin/GA ratios, the resulting conductive aerogels demonstrated similar (**a**) $\sigma_{30}$ and (**b**) $R_{0}$ values. Data are presented as mean ± s.d., *n* = 3, with each independent experiment marked by an open black circle. The above conductive aerogels demonstrated the same mixture loading of 10 mg mL^–1^. (**c**) By characterizing 3–4 replicates of conductive aerogels, measurement variations were observed in the $\sigma_{30}$ (~1.1 kPa) and $R_{0}$ (~10.1%) values. Error bars represent s.d.


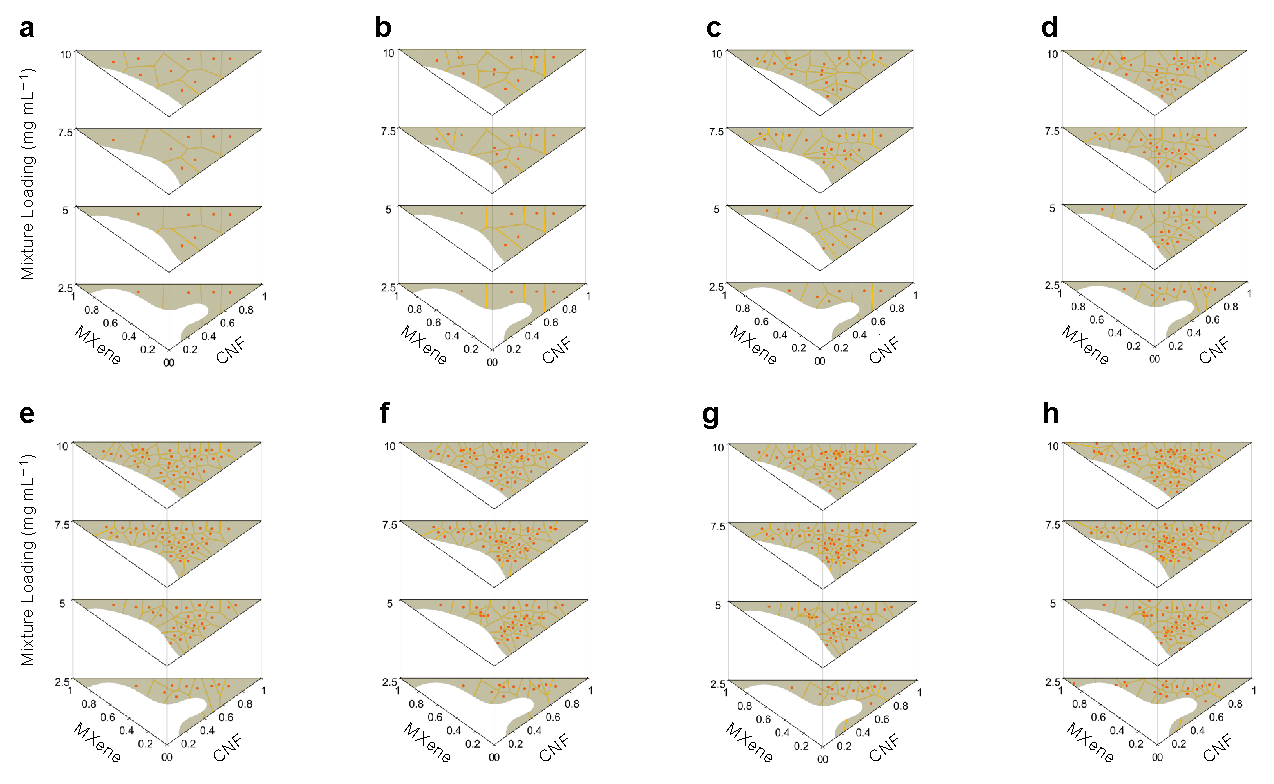
**Supplementary Fig. 12 2D Voronoi tessellation diagrams from loop #1 to loop #8.**


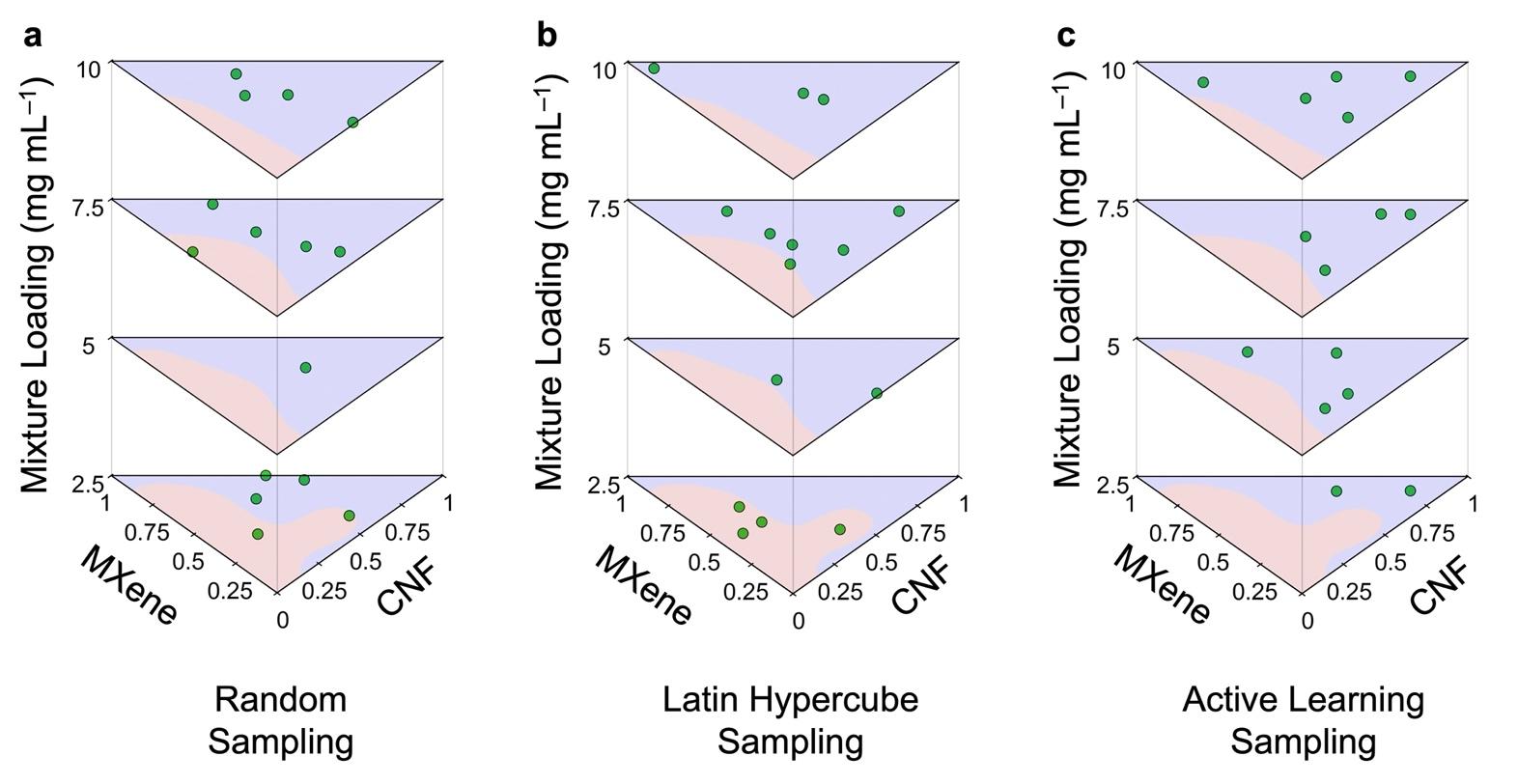


**Supplementary Fig. 13. Distribution profiles of data points collected from different sampling methods.** (a) Random sampling, (b) Latin hypercube sampling, and (c) active learning sampling. A total of 3 sampling cycles were performed, and 5 physical experiments were conducted in each cycle.


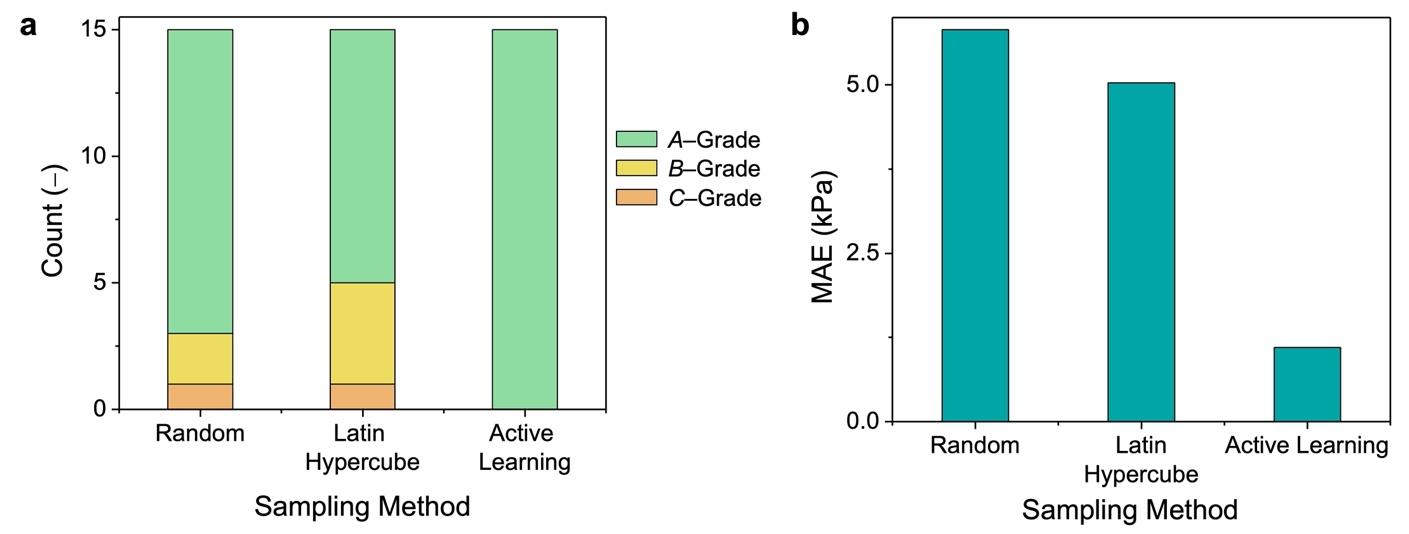


**Supplementary Fig. 14. Prediction model performance based on different sampling methods.** (a) Quantity of conductive aerogels at different grades based on different sampling methods. The active learning sampling was able to recommend the MXene/CNF/gelatin ratios with a >95% successful rate in producing A-grade aerogels. (b) MAE values of the prediction models based on different sampling methods. Trained by the data points collected from the active learning sampling, the prediction model demonstrated better learning efficiency and higher prediction accuracy, as evidenced by the lowest MAE of 1.1 kPa. Whereas the MAEs from random sampling and Latin hypercube sampling were 5.8 kPa and 5.0 kPa, respectively, after completing three cycles.

**
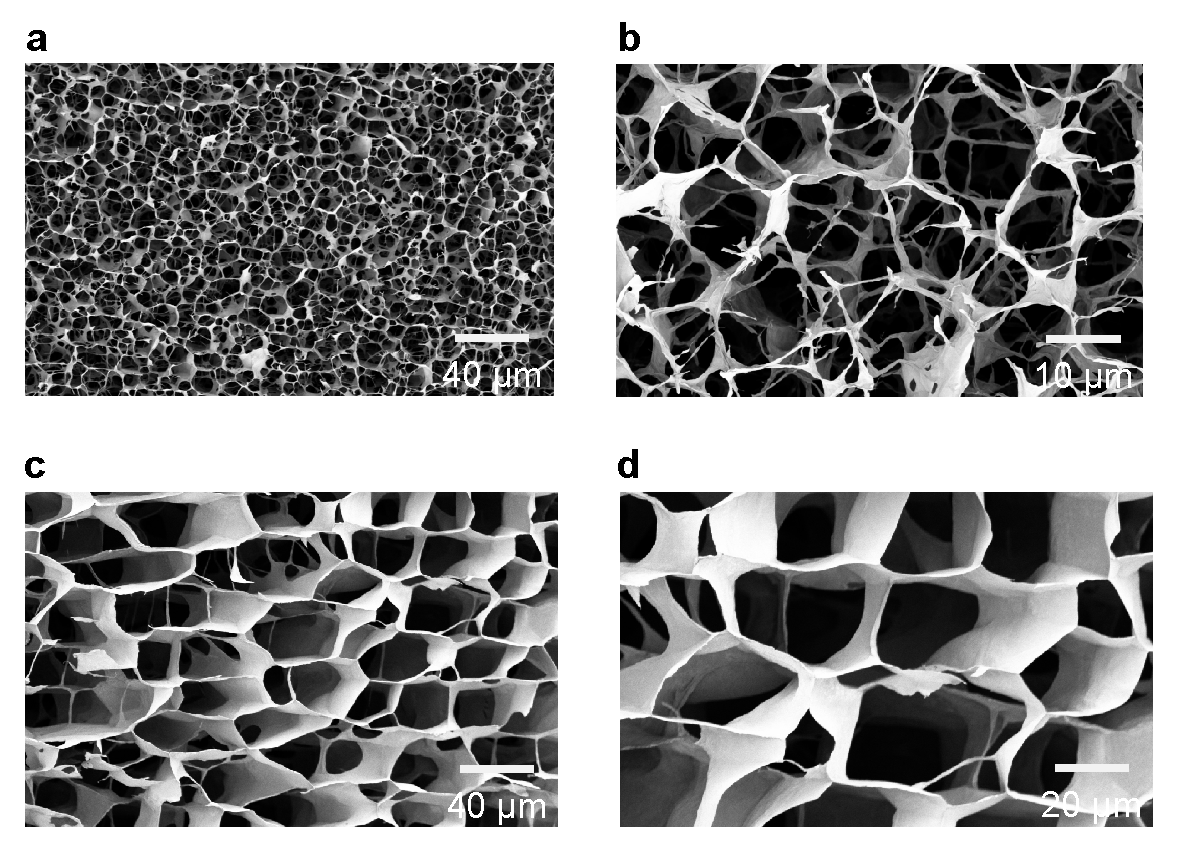
**

**Supplementary Fig. 15. SEM images of model-suggested conductive aerogels.** (**a**)(**b**) SEM images of a model-suggested conductive aerogel that met the design request #1. The design request #1 was $\sigma_{30}$ >10 kPa, and the champion model suggested recipe #9 (MXene/CNF/gelatin/GA ratio of 22/66/12/+ and mixture loading of 10.0 mg mL^–1^). (**c**)(**d**) SEM images of a model-suggested conductive aerogel that met the design request #2. The design request #2 was $\sigma_{30}$ >10 kPa and $R_{0}$ <10 Ω, and the champion model suggested recipe #14 (MXene/CNF/gelatin/GA ratio of 72/22/6/+ and mixture loading of 10.0 mg mL^–1^).

**
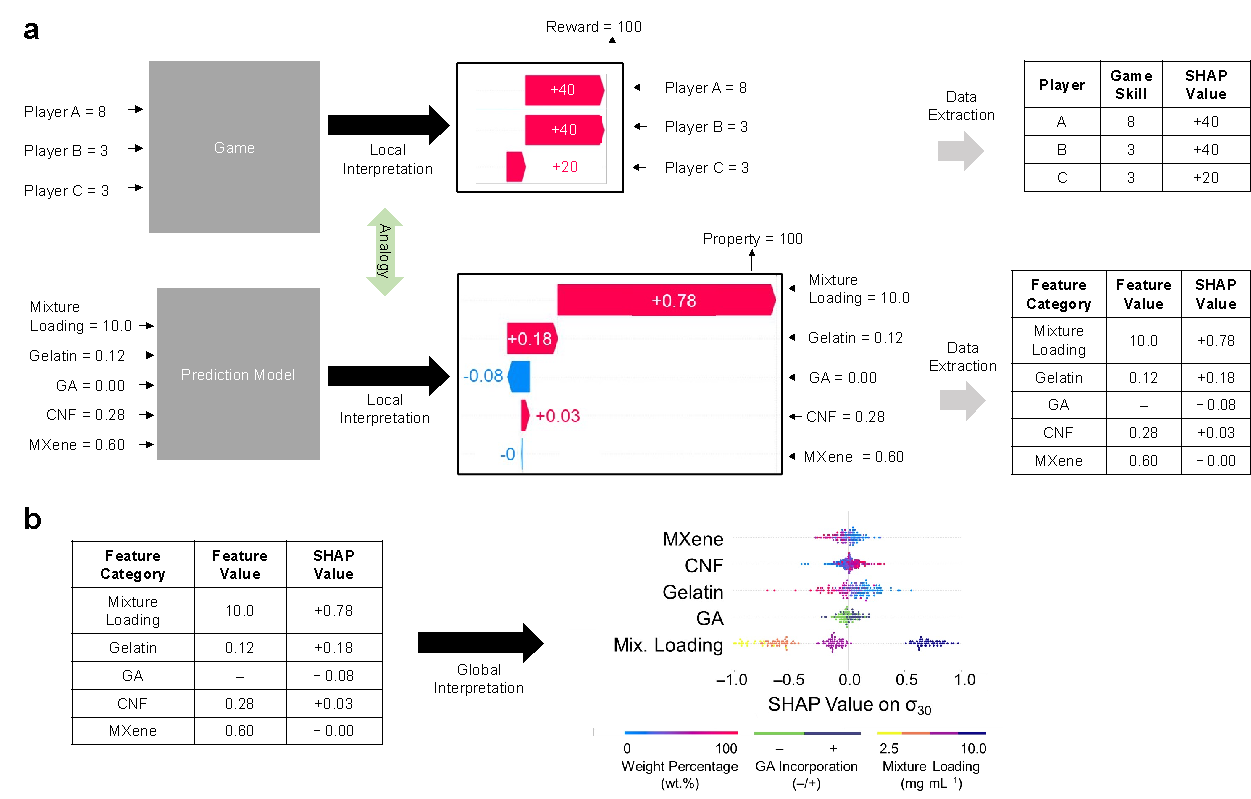
**

**Supplementary Fig. 16. Working mechanism of SHapley Additive exPlanations (SHAP).** (**a**) An analogy between the interpretation of a specific game and the interpretation of a prediction model. The red and blue bars represent the positive and negative SHAP values, respectively. (**b**) Figure plotting to get the global interpretation of the prediction model by using the SHAP values of every feature for every data point.

**
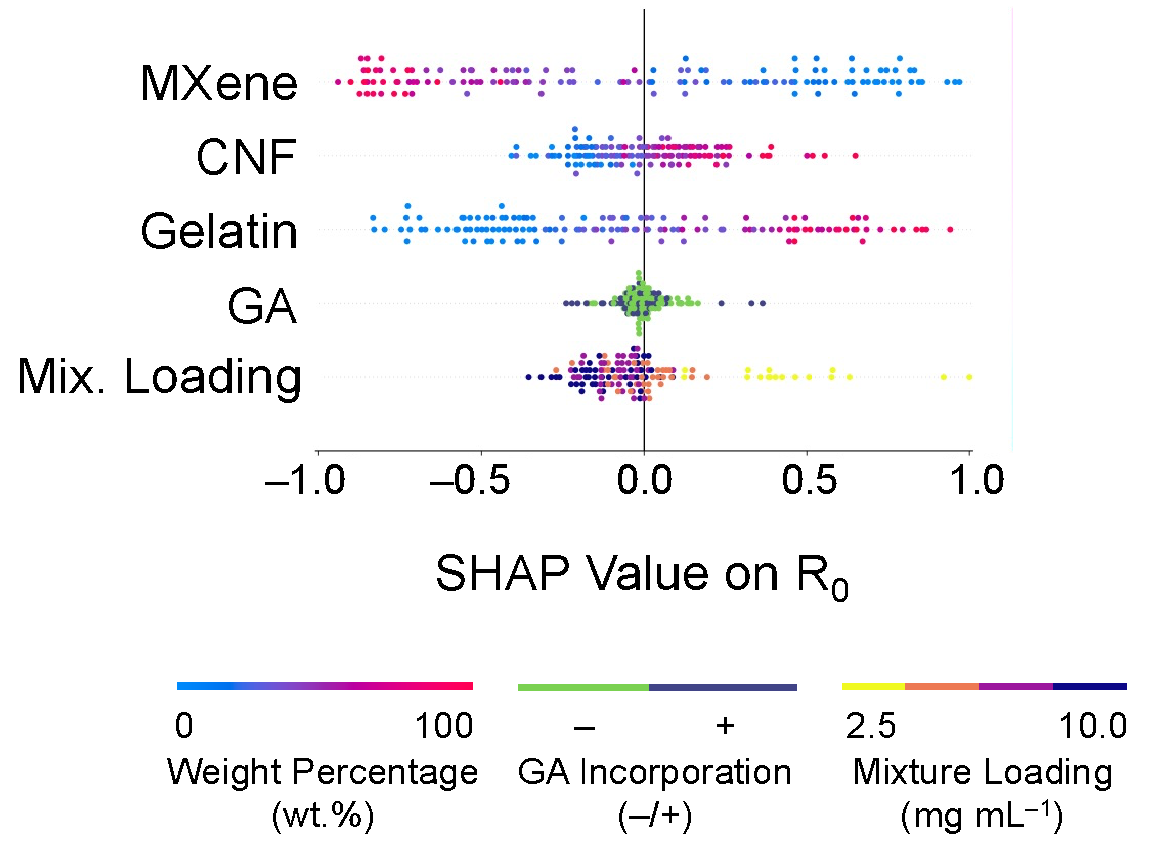
**

**Supplementary Fig. 17. SHAP values of MXene loading, CNF loading, gelatin loading, and GA loading, and mixture loading on the** $R_{0}$ **values of conductive aerogels.**

**
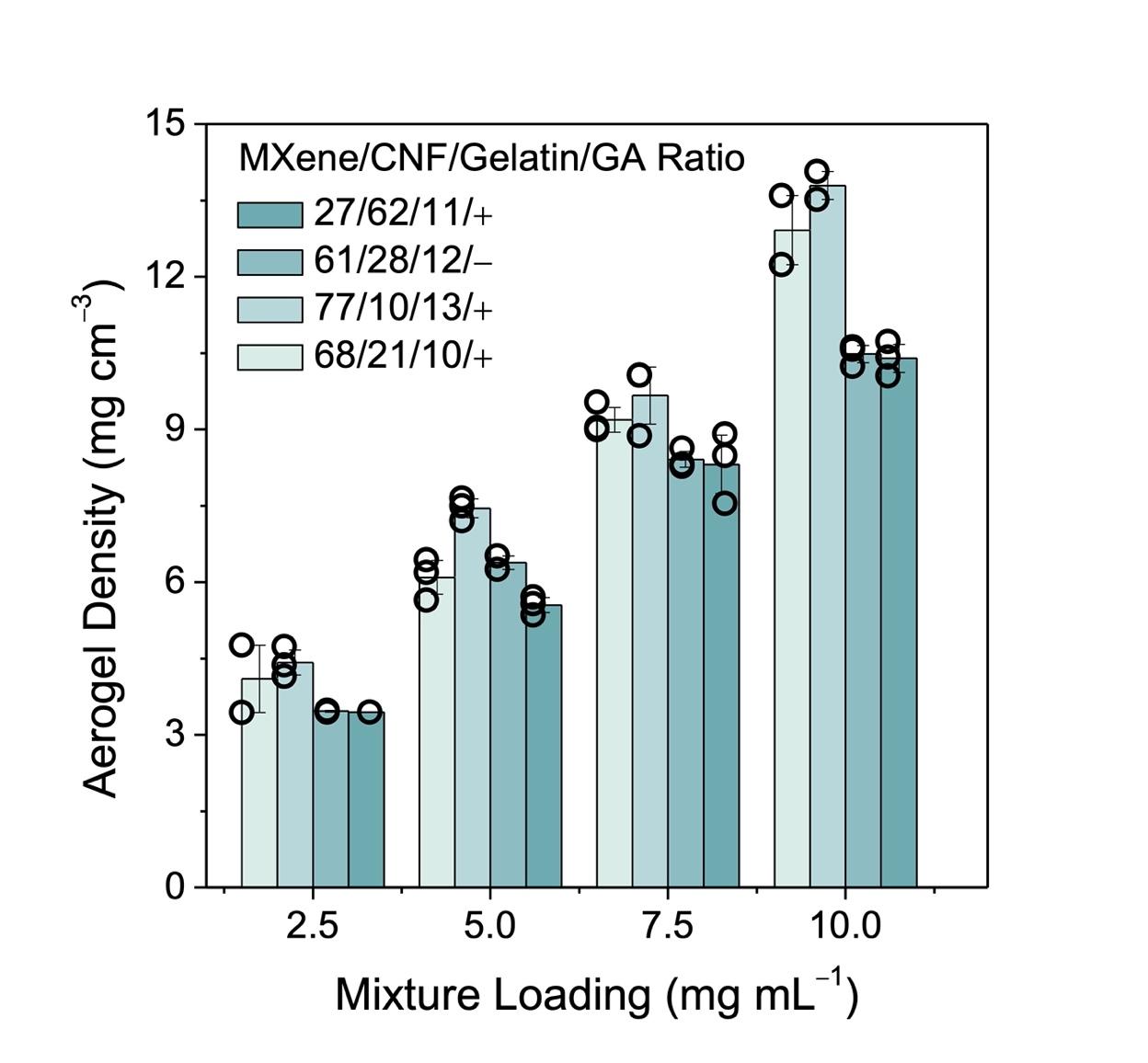
**

**Supplementary Fig. 18. Positive relationships between aerogel density and mixture loading.** We characterized the densities of conductive aerogels at four different MXene/CNF/gelatin/GA ratios and four mixture loadings, which ranged from 2.5 to 10.0 mg mL^–1^. Data are presented as mean ± s.d., *n* = 3, with each independent experiment marked by an open black circle. Error bars represent s.d.

**
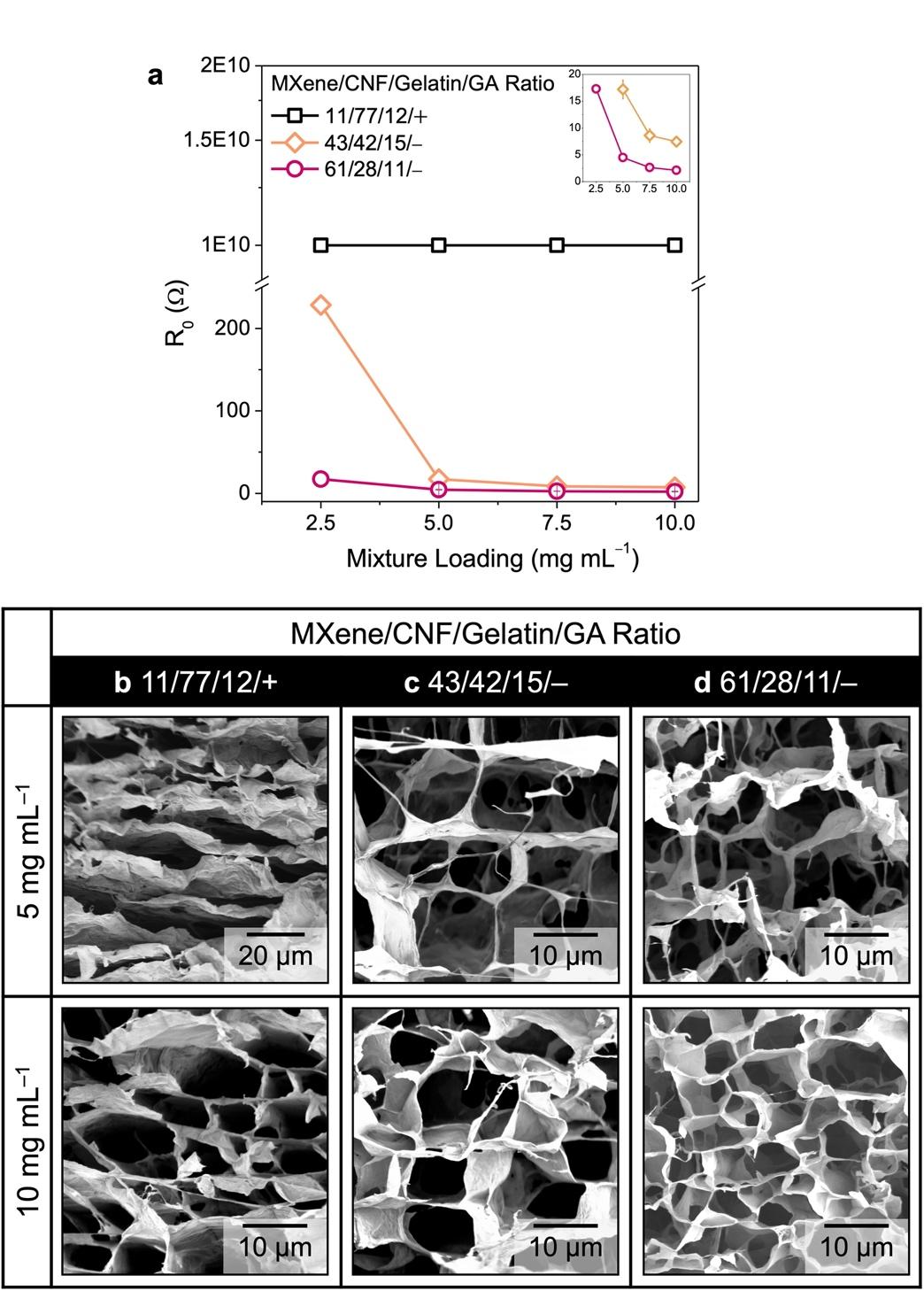
**

**Supplementary Fig. 19. Dependence of electrical resistances and aerogel microstructures on mixture loading.** (**a**) Correlations between aerogel electrical resistance and mixture loading. (**b**) SEM images of conductive aerogels at the MXene/CNF/gelatin/GA ratio of 11/77/12/+ at different mixture loadings. (c) SEM images of conductive aerogels at the MXene/CNF/gelatin/GA ratio of 61/28/11/– at different mixture loadings. (d) SEM images of conductive aerogels at the MXene/CNF/gelatin/GA ratio of 43/42/15/– at different mixture loadings.

**
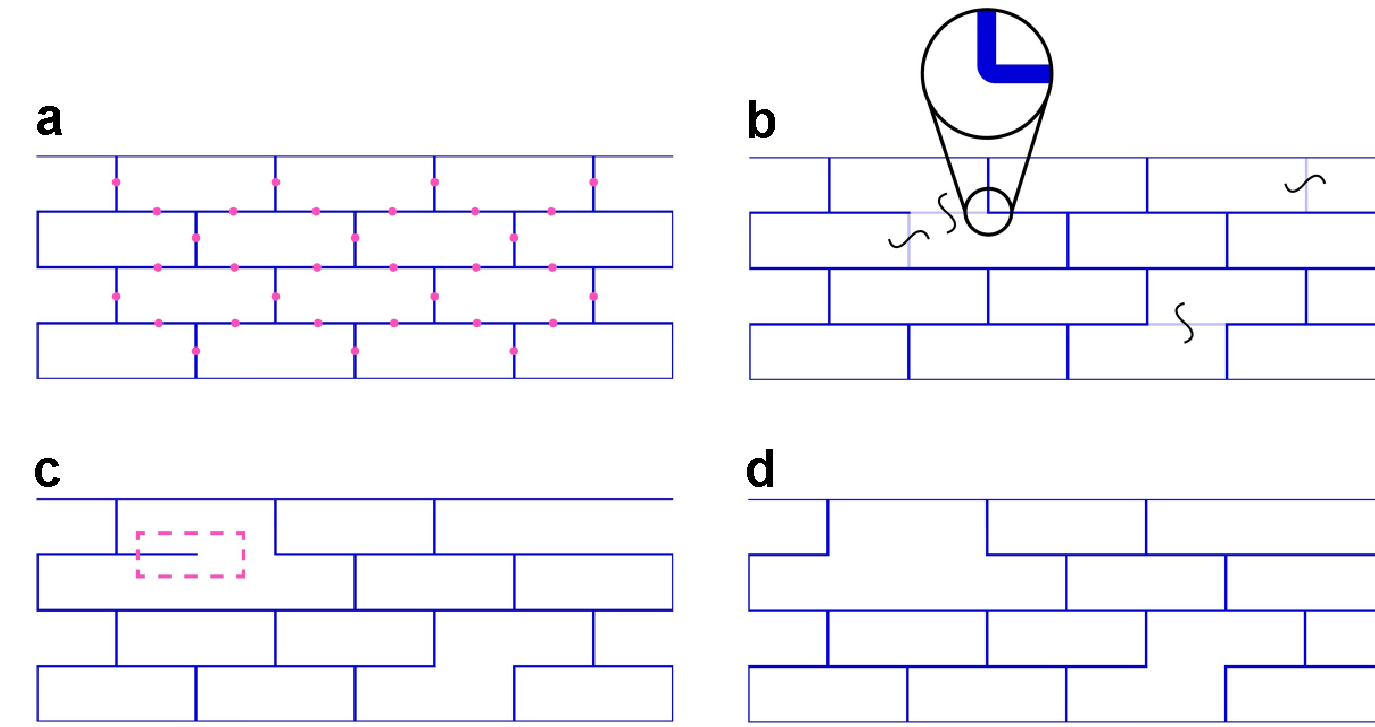
**

**Supplementary Fig. 20. Numerical geometries for FE models.** (**a**) Every wall segment within the lattice that could accommodate a valid discontinuity was cataloged into an array. (**b**) A random assortment of locations was selected to host wall discontinuities. At the designated locations, the wall segments were completely eliminated, leaving rounded corners at the adjoining wall intersections to facilitate contacts. (**c**) Independent wall sections that did not bear load and could potentially cause problems for simulated contact were removed. (**d**) The final geometry with random wall discontinuities was generated.

**
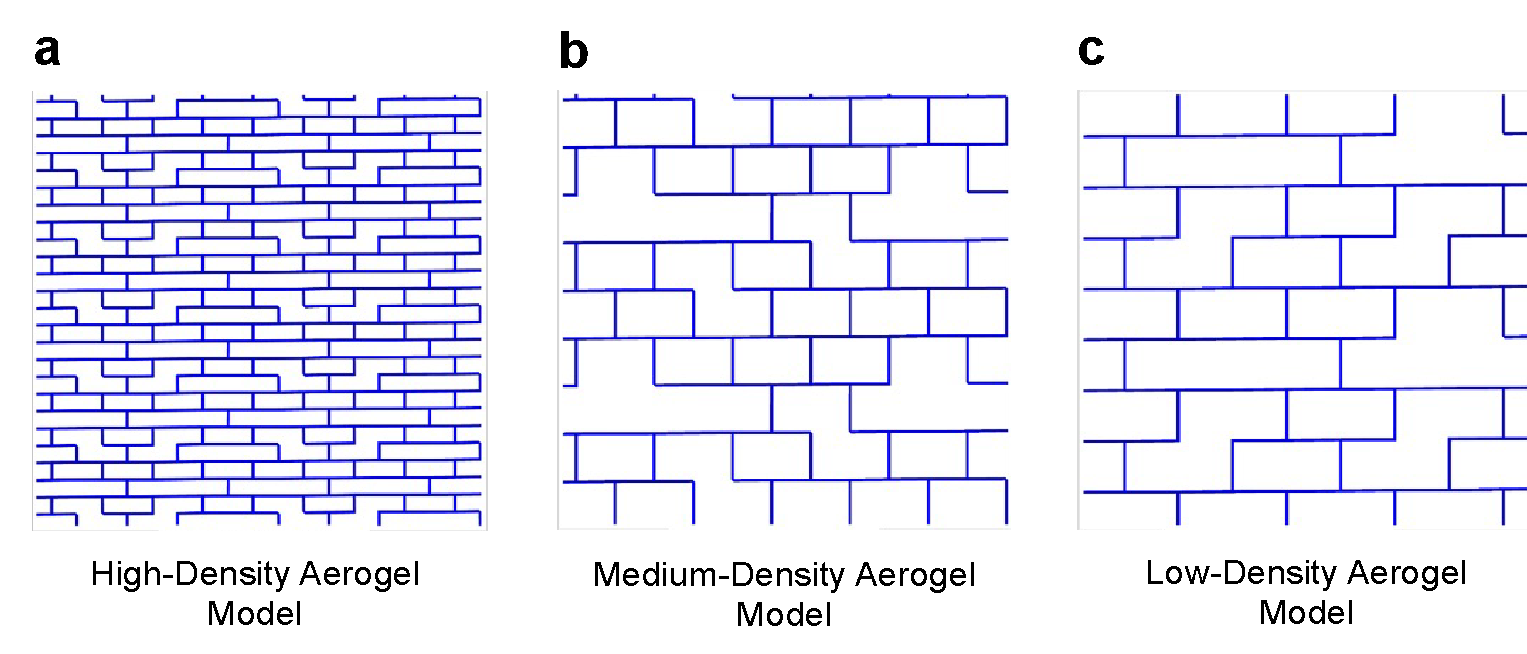
**

**Supplementary Fig. 21. High-, medium-, low-density aerogel models at their relaxed states.** (**a**) High-density aerogel model. (**b**) Medium-density aerogel model. (**c**) Low-density aerogel model.

**
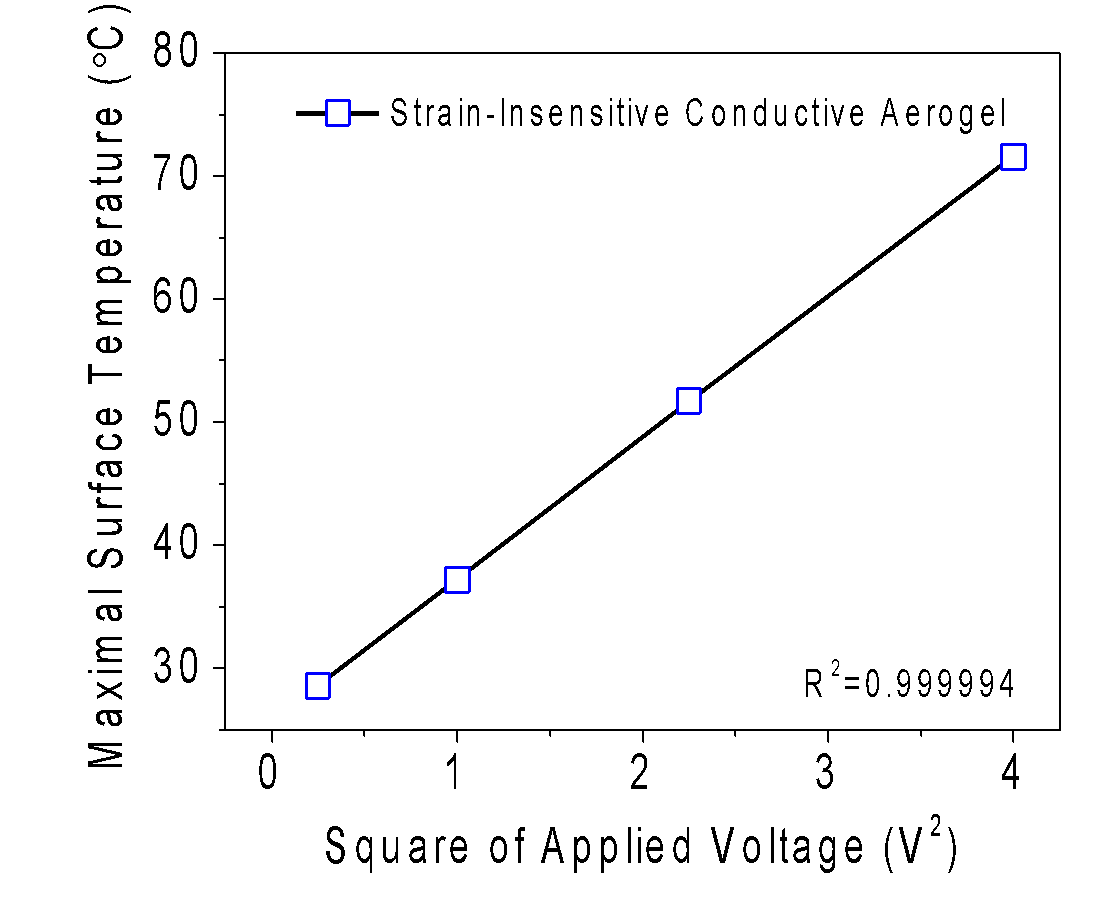
**

**Supplementary Fig. 22. Joule’s law of strain-insensitive conductive aerogels.** Maximal surface temperature of a strain-insensitive conductive aerogel (at the MXene/CNF/gelatin/GA ratio of 78/13/9/– and the mixture loading of 7.5 mg mL^–1^) were linearly correlated with the square of applied voltages.


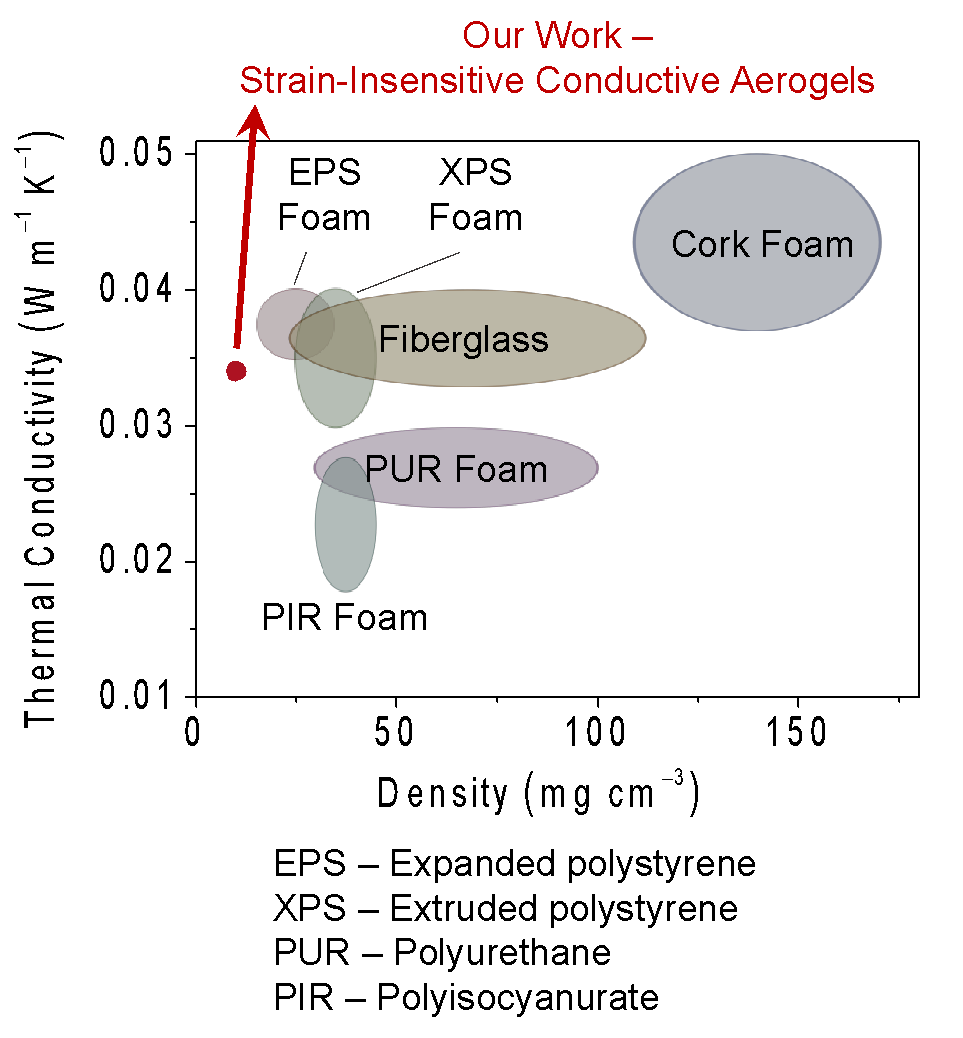


**Supplementary Fig. 23. Comparison of strain-insensitive conductive aerogels with commercial foam materials in terms of thermal conductivity and density.** The data of commercial foam materials are collected from the literature.^1^

**Supplementary Note1. Rationale of building block selection and model expansion strategy.**

The rationale behind selecting Ti_3_C_2_T_x_ MXene nanosheets, CNFs, and gelatin for the fabrication of conductive aerogels results from the synergistic effects of these building blocks on the aerogel’s properties.

1. Ti_3_C_2_T_x_ MXene nanosheets:

- Electrical conductivity: Ti_3_C_2_T_x_ MXene nanosheets, belonging to a class of two-dimensional materials, are known for their high electrical conductivity, which makes them a preferred choice for boosting the electrical conductivity of conductive aerogels.
- Surface chemistry: With abundant functional groups like –OH, –F, and –O on the MXene surfaces, MXene nanosheets can form stable interactions with other building blocks (such as CNFs and gelatin) and enhance the structural stability of the resulting aerogels.

1. Cellulose nanofibers (CNFs):

- Structural reinforcement: CNFs are renowned for their superior mechanical strength and stiffness, acting as reinforcement units in the conductive aerogels.
- High aspect ratio: The high aspect ratio of CNFs can facilitate the formation of a percolated network throughout the conductive aerogel, leading to enhanced mechanical resilience.
- Hydrophilicity: The inherent hydrophilicity of CNFs aids in the uniform distribution of other building blocks within the aerogel structure.

1. Gelatin:

- Gelling agent: Gelatin, a natural biopolymer, excels as a gelling agent, especially when combined with crosslinking agents, such as glutaraldehyde.
- Affinity to other building blocks: Because of its ability to form robust molecular interactions, gelatin can bridge MXenes and CNFs effectively, creating potential hybrid systems of conductive aerogels.

The selection of building blocks is pivotal for defining the functional capabilities of lightweight aerogels. For instance, it is advantageous to integrate more natural components into the parameter space of multi-component aerogels, such as gellan gum, polyvinyl alcohol (PVA), and sodium alginate. When composed at varying ratios, these aerogels can be rehydrated to create a variety of functional hydrogels suitable for agricultural applications, including serving as growth media for microgreens and baby greens (see Supplementary Fig. 2a, b). To enhance the prediction model with a broader range of building blocks, it is advisable to adopt a model expansion method to augment its predictive power. As shown in Supplementary Fig. 2c, additional active learning loops should be performed under the guidance of the prediction model. During the model expansion phase, additional experiments are required to refine the SVM classifier and to update the ANN-based model. By strategically selecting new components in tandem with the model expansion method, the prediction model can consistently enlarge its parameter space and broaden the range of achievable functions. However, this model expansion incurs additional active learning cycles, leading to higher time and cost implications. To mitigate this, the development of automated fabrication and characterization platforms is essential to enhance data collection efficiency and lessen human labor demands. Additionally, establishing an open database would offer substantial benefits to the wider research community.

**Supplementary Note 2. Estimated number of experiments required to build an extensive dataset for conductive aerogels.**

To compare our ML framework with the one-factor-at-a-time (OFAT) design of experiment (DoE) method, we calculated the number of data points needed to cover the entire design space using Supplementary Equations 1 and 2,

$S_{x}=\frac{\left( n! \right)}{k!\cdot\left( x-1 \right)!}$ (Supplementary Equation 1)

$n=x-1+k$ (Supplementary Equation 2)

, where $S_{x}$ denotes the number of data points, $x$ is the number of DOFs, and $1/k$ is the step size. The Python code is available on GitHub at <https://github.com/oshin71/Programmable-MXene-Aerogel/blob/main>.

For the composition labels, three DOFs ($x=3$) are identified for the fabrication of conductive aerogels. For the loading label of conductive aerogels, one DOF is identified. Considering a step size of 2.0 wt.% for the MXene, CNF, and gelatin loadings ($\frac{1}{k}=$0.02), two options (+/–) for the GA incorporation, and four mixture loadings, a total number of experiments required to construct an extensive dataset is estimated to be 5,300. In addition, two property labels, including $\sigma_{30}$ and $R_{0}$, need to be collected for each conductive aerogel. Using the step size of 2.0 wt.%, the OFAT DoE method requires 5,300 data points. As depicted in Supplementary Fig. 6, the number of data points is inversely correlated with the step size.

**Supplementary Note 3. Multi-stage AI/ML framework.**

We provide in-depth justifications for the algorithmic choices in the AL/ML framework and elucidate the reasons why the selected algorithms outperform traditional or simplistic methods in terms of efficiency, accuracy, and scalability.

**SVM classifier:** Within the AI/ML framework, the SVM classifier acted as a crucial filtering unit for the prediction model, ensuring the model-suggested MXene/CNF/gelatin/GA ratios that produced A-grade conductive aerogels. In contrast to other systems rich in data, the fabrication of conductive aerogels is time-consuming and labor-intensive. In the absence of the SVM classifier, the prediction model could suggest the MXene/CNF/gelatin/GA ratios that result in lower-grade conductive aerogels, which would not be efficient for collecting high-quality data points. Therefore, a high-accuracy SVM classifier is critical to ensure the consistent production of A-grade conductive aerogels throughout the all the active learning loops.

**ANN committee:** As indicated in Fig. 2e, the prediction models based on alternative algorithms struggled to accurately predict the $\sigma_{30}$ and $R_{0}$ values of conductive aerogels based on their MXene/CNF/gelatin/GA ratios and mixture loadings. In contrast, our prediction model, which incorporated an ANN committee, demonstrated the lowest MAE of 1.5 kPa (for $\sigma_{30}$) and MRE of 18.4% (for $R_{0}$) after the active learning loops. These results clearly show that the ANN committee is required to accurately predict the mechanical and electrical properties of conductive aerogels in a complex, non-linear system with multiple DOFs.

**Data augmentation:** To overcome the overfitting challenges upon the use of a small dataset, we employed a data augmentation method, specifically the UIP method, to synthesize virtual data points in the vicinity of real data points collected during the active learning loops. By feeding both real and virtual data points into the ANN committee, the prediction model demonstrated higher predictive accuracy, as evidenced by lower MRE and MAE values, as demonstrated in Fig. 2f**.**

In conclusion, the multi-stage AI/ML framework, composed of an SVM classifier, active learning loops (incorporating an ANN committee), and data augmentation, can synergistically address the data scarcity challenges encountered in the field of conductive aerogels, where the collection of high-quality data points is labor-intensive and time-consuming.

**Supplementary Note 4. Classification standards used for categorizing 264 samples.**

Discontinuities in the aerogel’s network can severely compromise its strength, structure, and functionality. The presence of cracks, deformities, and a lack of cohesion can lead to the aerogel breakage into irregular pieces or total pore structure collapse. For the SVM classifier aimed at predicting high-quality conductive aerogels, rigorous classification standards were established.

A-grade conductive aerogel standard: Monolithic, freestanding conductive aerogels with minimal imperfections and limited fragmentation are classified as A-grade. Aerogel samples with minor deformities, especially if localized around the edges, are also classified as A-grade. Conductive aerogels with fragments can also be designated as A-grade if they retain at least 80% of their original volume and sustain a regular structure with a uniform base and height.

B-grade conductive aerogel standard: Conductive aerogels fractured into several, yet substantial fragments, fall under B-grade. Although these fragments are often monolithic, freestanding, and porous, their varied shapes and sizes make them less reliable for precise mechanical and resistance measurements.

C-grade conductive aerogel standard: Highly deformed or collapsed conductive aerogel samples are categorized as C-grade. These samples present a loose structure, lacking the ability to stand freely.

**Supplementary Note 5. Training of a SVM classifier.**

To facilitate the prediction model to suggest a set of suitable fabrication parameters for A-grade conductive aerogels, a high-accuracy SVM classifier was trained. The model training was achieved through a two-step process, including (1) selecting a suitable kernel function and (2) optimizing SVM hyperparameters. Given that the collected grades exhibited non-linear properties, we opted to use a kernel function that mapped discrete data points into a higher dimensional feature space, thereby facilitating the identification of optimal hyperplanes with maximal margin distances. We selected the Radial Basis Function (RBF) as our kernel function to handle the non-linear data points. Subsequently, we implemented Bayesian optimization with a 4-fold cross validation process to fine-tune the hyperparameter values. The SVM classifier was trained using 264 grades, which achieved a prediction accuracy of 95% based on 40 testing data points. We have recorded the Python code used for training the SVM model available on GitHub for open-source use. It can be accessed at <https://github.com/oshin71/Programmable-MXene-Aerogel/blob/main/design_boundary.ipynb>.

**Supplementary Note 6. Implementation of UIP method.**

The utilization of a small dataset can potentially lead to model overfitting issues. To address this challenge, we have employed a well-known method, the UIP method, to augment the data points collected during the active learning loops. The UIP method is based on the natural principles proposed by expert users. For instance, it is observed that the property labels of a conductive aerogel remain approximately constant when there are slight variations in specific composition labels. As depicted in Supplementary Fig. 11a, b, when the MXene/CNF/gelatin/GA ratio slightly changed from 64/24/12/+ to 62/26/12/+, the resulting conductive aerogels demonstrated similar $\sigma_{30}$ and $R_{0}$ values. Moreover, we note that there are some measurement variations in the $\sigma_{30}$ and $R_{0}$ values. As illustrated in Supplementary Fig. 11c, by charactering 3–4 conductive aerogel replicates (following the same fabrication parameters), the $\sigma_{30}$ and $R_{0}$ values presented the measurement variations of ~1.1 kPa and ~10.1%, respectively. In this study, the UIP method was adopted to synthesize 1,000-fold virtual data points by introducing Gaussian noises in the vicinity of 162 real data points collected during the active learning loops. We have recorded the Python code used for implementing the UIP method available on GitHub for open-source use. It can be accessed at <https://github.com/oshin71/Programmable-MXene-Aerogel/blob/main/data_augmentation.ipynb>.

**Supplementary Note 7. Calculation of A Score acquisition function.**

A suitable acquisition function was introduced in the active learning loops to suggest the targeted data points with the highest uncertainty in the feasible design space. We defined an acquisition function as A Score in Supplementary Equation 3,

$A Score=L_{2}\cdot\hat{\sigma}$ (Supplementary Equation 3)

, where $L_{2}$ denotes the shortest mathematical distance (also called Euclidian distance) between current composition labels (within the dataset of prediction model) and targeted composition labels (not yet included in the dataset of prediction model). In particular, $L_{2}$ is calculated by Supplementary Equation S4,

$L_{2}=\sqrt{\min_{i \in N}\left[ \left( \left[ \mathrm{MXene}_{i} \mathrm{CNF}_{i} \mathrm{GEL}_{i} {GA}_{i} {\mathrm{Mixture} \mathrm{Loading}}_{i} \right]-\left[ \mathrm{MXene}_{j} \mathrm{CNF}_{j} \mathrm{GEL}_{j} {GA}_{j} {\mathrm{Mixture} \mathrm{Loading}}_{j} \right] \right)^{2} \right]}$ (Supplementary Equation 4)

, where *N* is the cumulative number of data points in current dataset, $\mathrm{MXene}_{i}$, $\mathrm{CNF}_{i}$, $\mathrm{GEL}_{i}$, $\mathrm{GA}_{i}$, and ${\mathrm{Mixture} \mathrm{Loading}}_{i}$ represent the MXene, CNF, gelatin, GA loadings, and the mixture loading of one known data point (*i*) (already in the database), and $\mathrm{MXene}_{j}$, $\mathrm{CNF}_{j}$, $\mathrm{GEL}_{j}$, $\mathrm{GA}_{j}$, and ${\mathrm{Mixture} \mathrm{Loading}}_{j}$ are the labels of one targeted data point (*j*) (in the feasible parameter space yet not included in the database).

On the other hand, $\hat{\sigma}$ denotes the variance of predicted property labels from the ANN committee, which is defined in Supplementary Equation S5,

$\hat{\sigma}=\sqrt{\frac{1}{M}\sum_{j=1}^{M} \left( \left[ \mathrm{Output}_{\sigma_{30}}^{m} \mathrm{Output}_{R_{0}}^{m} \right]-\left[ \left[ \mathrm{Output}_{\sigma_{30}}^{\mathrm{Ave}} \mathrm{Output}_{R_{0}}^{\mathrm{Ave}} \right] \right] \right)^{2}}$ (Supplementary Equation 5)

, where $M$ is the number of ANN programs in the model committee ($M$ = 5), $\mathrm{Output}_{\sigma_{30}}^{m}$ and $\mathrm{Output}_{R_{0}}^{m}$ are the output $\sigma_{30}$ and $R_{0}$ values predicted by the *m*^th^ decision program, $\mathrm{Output}_{\sigma_{30}}^{Ave}$ and $\mathrm{Output}_{R_{0}}^{Ave}$ are the average $\sigma_{30}$ and $R_{0}$ values predicted by the ANN committee. We have recorded the Python code used for implementing A Score-based active learning loops available on GitHub for open-source use. It can be accessed at the following link: <https://github.com/oshin71/Programmable-MXene-Aerogel/blob/main/active_leaerning.ipynb>.

**Supplementary Note 8. Shapley Additive exPlanations (SHAP) model interpretation method.**

The SHAP analysis is a game theoretic approach to explain the output of any ML model (including the ensemble models). It can find the feature of importance inside the ML models, thus enabling the users to interpret the models. The process to find the feature importance is like finding the contribution of each player in a collaborative game.

To understand how the feature importance is found in SHAP, an example is presented below. As shown in Supplementary Fig. 16a, there are three game players (i.e., A, B, and C). They collaborate with each other to play a game. When all of them join the game, based on their different skills in a specific game (e.g., 8, 3, and 3 for players A, B, and C.), they can achieve a 100 reward. The task is to quantify how important each player is in getting the reward. To solve this, we assume that the game players join in a specific sequence, (e.g., player A first, then player B, next player C) and the marginal rewards of each player are recorded. For example, player A is the first member with a reward of 40, then player B joins the game and brings the reward to 80, and next player C joins to bring the reward to 100. Therefore, the players’ respective marginal rewards are “player A, B, C = 40, 40, 20”. However, the calculated marginal reward may not accurately represent the contribution of each player. For example, when the sequence is changed from player A, B, C to player A, C, B, if players B and C have a similar skill set, the rewards are still 40, 40, and 20. Then the players’ respective marginal rewards are changed to “player A, B, C = 40, 20, 40”. Therefore, the sequence of how the players join the game is important.

In order to get a more accurate reward of each individual player, we need to find out the marginal reward of each player under every possible sequence. The reward for each individual player then can be the sum of these marginal rewards over the number of possible sequences (the calculation for a specific player is illustrated in Supplementary Equation S6). For example, in the case outlined above, we can simulate the entering sequences: ABC, ACB, BCA, BAC, CAB, and CBA, and the marginal reward of each player is recorded for each sequence. Then, by averaging all of these rewards, we obtain the reward contributed from each player. This reward is the SHAP value.

$SHAP value of a player = \frac{Sum of mariginal rewards of a specific player under all possible sequences}{Number of total possible sequences}$ (Supplementary Equation 6)

Back to the feature importance analysis of the developed champion model, we can take the problem as an analogy to the above case. All composition and loading labels (i.e., MXene, CNF, gelatin, GA loadings, and mixture loading) are regarded as players, which are fed into the prediction model to obtain the property labels. The prediction process is treated as the game, and the deviation (between the predicted property label of a specific data point and the average property label from all data points) is treated as the reward. By following Fig **S7**, the SHAP value of each composition label on a specific property label can be calculated, and this value is used to measure the feature importance.

$SHAP value of a composition/loading label = \frac{Sum of mariginal reward of a compoisiton/loading label on a specific property label under all possible sequences}{Number of total possible sequences}$ (Supplementary Equation **7**)

The above process is the interpretation of the prediction model on a specific data point which is called the local interpretation. To get the global interpretation of the prediction model over all data points, we can plot the SHAP values of every composition or loading label for every data point (Supplementary Fig. 16b). A wider range of the SHAP value for a specific feature indicates a higher importance, and *vice versa*. We have recorded the Python code used for implementing SHAP analyses available on GitHub for open-source use. It can be accessed at <https://github.com/oshin71/Programmable-MXene-Aerogel/blob/main/plot_shaps.ipynb>.

**Supplementary Note 9. FE model construction for conductive aerogels.**

To gather a better understanding of the mechanical behaviors of conductive aerogels at different mixture loadings, we performed FE simulations with the commercially available software ABAQUS/CAE 2020. In the FE simulations, the cellular structure of a conductive aerogel was represented using a two-dimensional staggered brick-wall architecture, whose dimensions were extracted from the SEM images in Fig. 4c. Periodic boundary conditions were imposed to mimic the periodic nature and to mitigate the Impact of boundary effects on the numerical results.^2^ Based on the SEM images of conductive aerogels, analogous random discontinuities were seeded within the FE models. A 4 × 4 super cell was chosen to encapsulate a large enough bound for varying locations of seeded discontinuities, while it remained small enough to minimize computing time. The wall of conductive aerogels was modeled as an isotropic material, whose mechanical properties were acquired experimentally (see Supplementary Table 9). In the super cell, the aerogel structure was meshed using plane strain 8-node biquadratic hybrid elements with reduced integration (ABAQUS element type CPE8RH) with second-order accuracy, and the mesh convergence has been verified. We conducted a static analysis (*STATIC step with NLGEOM=ON in ABAQUS) by linearly increasing the vertical displacement upon the compressive vertical strain of 30%, from which the $\sigma_{30}$ values of conductive aerogels were derived and compared with the experimental results.

To capture the wall discontinuities of conductive aerogels, random discontinuities were seeded in the periodic structure of a FE model, based on the following method:

1. Possible discontinuity locations were created within the supercell, as shown in Supplementary Fig. 20a.
2. A number of unit cells within the supercell were randomly chosen to host an imperfection based on a tuning parameter (Supplementary Equation S8),

$\beta_{imp}=\frac{n_{i}}{N_{s}}$ (Supplementary Equation 8)

, where $n_{i}$ represents the number of imperfect cells within the supercell and $N_{s}$ represents the total number of cells of the supercell. For each imperfect unit cell, a wall imperfection was seeded at a random location, and sharp corners were rounded to facilitate contacts, as shown in Supplementary Fig. 20b.

1. Floating wall segments were removed to facilitate contact within the FE simulations, as shown in Supplementary Fig. 20c.
2. Hard, frictionless surface-to-surface self-contacts were imposed in the aerogel structure to finalize the model construction process, as shown in Supplementary Fig. 20d.

For all three FE models, the same $\beta_{imp}$of 0.3 was considered. Based on the first buckling mode of aerogel structures, a nodal displacement of 5% was imposed to the randomly imperfect the FE models. FE simulations were performed for three conductive aerogels at different mixture loadings (i.e., 5.0, 7.5, and 10.0 mg mL^–1^) to construct high-, medium-, and low-density aerogel models, respectively. By exerting a vertical compressive strain of 30% on three FE models, the $\sigma_{30}$ values were simulated in Fig. 4b, showing good agreement with the experimentally characterized $\sigma_{30}$ values.

To simulate the microstructure of the conductive aerogels under compression, two different physical parameters were analyzed from the SEM images in Fig. 4c, including pore density (ρ) and pore aspect ratio ($\psi$). The $\psi$ value was characterized by the pore shape. As depicted in Supplementary Fig. 21a**–c**, the ρ of each aerogel model was quantified using Supplementary Equation S9,

$\rho=\frac{n_{m}}{N_{t}^{p}}$ (Supplementary Equation 9)

, where $n_{m}$ is the number of pixels of aerogel model and $N_{t}^{p}$ is the total number of pixels of the 4 × 4 supercells. Similarly, the $\psi$ of each aerogel model was calculated using Supplementary Equation S10,

$\psi=\frac{w}{h}$ (Supplementary Equation 10)

, where $w$ and $h$ are the cell width and height, respectively (see Supplementary Table 9). The $\rho$ and $\psi$ values of high-, medium-, and low-density aerogel models are presented in Supplementary Table 9.

**Supplementary Table 1. Description of independent and dependent variables.**

| **Independent Variable** | **Unit** | **Physical Meaning Description** |
| --- | --- | --- |
| MXene Loading | wt.% | Weight percentage of MXene nanosheets in an aqueous mixture |
| CNF Loading | wt.% | Weight percentage of CNFs in an aqueous mixture |
| Gelatin Loading | wt.% | Weight percentage of gelatin in an aqueous mixture |
| GA Loading | +/– | Options of adding GA into an aqueous mixture  (“+” with GA addition; “–” without GA addition) |
| Mixture Loading | mg mL^–1^ | Solid content in an aqueous mixture |
|  | | |
| **Dependent Variable** | **Unit** | **Physical Meaning Description** |
| $R_{0}$ | Ω | Initial electrical resistance under 0% strain |
| $\sigma_{30}$ | kPa | Compressive stress under 30% strain* |

*There are two reasons of selecting $\sigma_{30}$ as one of the dependent variables. First, several low-density aerogels were too fragile and could not sustain the compressive strains over 30%. Second, as shown in Supplementary Fig. 7, taking multiple points along the entire stress–strain curves as the training data points led to a prediction model with a higher MAE value of 4.5 kPa. On the other hand, when the value was used as the mechanical labels for training data points, the prediction model demonstrated a lower MAE value of 1.1 kPa.

**Supplementary Table 2. Discrete grades of 264 conductive aerogels with different MXene/CNF/gelatin ratios.** “A-grades” refer to large, intact conductive aerogels or sizable but fragmented aerogels. “B-grades” refer to small and fragmented aerogels. “C-grades” refer to extensively altered forms with inconsistent pieces.

| ID (–) | MXene (wt.%) | CNF (wt.%) | Gelatin (wt.%) | Mixture Loading (mg mL^–1^) | Grade (A/B/C) |
| --- | --- | --- | --- | --- | --- |
| 1 | 0 | 0 | 100 | 10.0 | A |
| 2 | 20 | 0 | 80 | 10.0 | A |
| 3 | 40 | 0 | 60 | 10.0 | B |
| 4 | 60 | 0 | 40 | 10.0 | B |
| 5 | 80 | 0 | 20 | 10.0 | A |
| 6 | 100 | 0 | 0 | 10.0 | A |
| 7 | 0 | 20 | 80 | 10.0 | A |
| 8 | 20 | 20 | 60 | 10.0 | A |
| 9 | 40 | 20 | 40 | 10.0 | A |
| 10 | 60 | 20 | 20 | 10.0 | A |
| 11 | 80 | 20 | 0 | 10.0 | A |
| 12 | 0 | 40 | 60 | 10.0 | A |
| 13 | 20 | 40 | 40 | 10.0 | A |
| 14 | 40 | 40 | 20 | 10.0 | A |
| 15 | 60 | 40 | 0 | 10.0 | A |
| 16 | 0 | 60 | 40 | 10.0 | A |
| 17 | 20 | 60 | 20 | 10.0 | A |
| 18 | 40 | 60 | 0 | 10.0 | A |
| 19 | 0 | 80 | 20 | 10.0 | A |
| 20 | 20 | 80 | 0 | 10.0 | A |
| 21 | 0 | 100 | 0 | 10.0 | A |
| 22 | 10 | 0 | 90 | 10.0 | A |
| 23 | 30 | 0 | 70 | 10.0 | B |
| 24 | 50 | 0 | 50 | 10.0 | B |
| 25 | 70 | 0 | 30 | 10.0 | C |
| 26 | 90 | 0 | 10 | 10.0 | A |
| 27 | 0 | 10 | 90 | 10.0 | B |
| 28 | 10 | 10 | 80 | 10.0 | B |
| 29 | 20 | 10 | 70 | 10.0 | B |
| 30 | 30 | 10 | 60 | 10.0 | B |
| 31 | 40 | 10 | 50 | 10.0 | B |
| 32 | 50 | 10 | 40 | 10.0 | A |
| 33 | 60 | 10 | 30 | 10.0 | A |
| 34 | 70 | 10 | 20 | 10.0 | A |
| 35 | 80 | 10 | 10 | 10.0 | A |
| 36 | 90 | 10 | 0 | 10.0 | A |
| 37 | 10 | 20 | 70 | 10.0 | A |
| 38 | 30 | 20 | 50 | 10.0 | A |
| 39 | 50 | 20 | 30 | 10.0 | A |
| 40 | 70 | 20 | 10 | 10.0 | A |
| 41 | 0 | 30 | 70 | 10.0 | A |
| 42 | 10 | 30 | 60 | 10.0 | A |
| 43 | 20 | 30 | 50 | 10.0 | A |
| 44 | 30 | 30 | 40 | 10.0 | A |
| 45 | 40 | 30 | 30 | 10.0 | A |
| 46 | 50 | 30 | 20 | 10.0 | A |
| 47 | 60 | 30 | 10 | 10.0 | A |
| 48 | 70 | 30 | 0 | 10.0 | A |
| 49 | 10 | 40 | 50 | 10.0 | A |
| 50 | 30 | 40 | 30 | 10.0 | A |
| 51 | 50 | 40 | 10 | 10.0 | A |
| 52 | 0 | 50 | 50 | 10.0 | A |
| 53 | 10 | 50 | 40 | 10.0 | A |
| 54 | 20 | 50 | 30 | 10.0 | A |
| 55 | 30 | 50 | 20 | 10.0 | A |
| 56 | 40 | 50 | 10 | 10.0 | A |
| 57 | 50 | 50 | 0 | 10.0 | A |
| 58 | 10 | 60 | 30 | 10.0 | A |
| 59 | 30 | 60 | 10 | 10.0 | A |
| 60 | 0 | 70 | 30 | 10.0 | A |
| 61 | 10 | 70 | 20 | 10.0 | A |
| 62 | 20 | 70 | 10 | 10.0 | A |
| 63 | 30 | 70 | 0 | 10.0 | A |
| 64 | 10 | 80 | 10 | 10.0 | A |
| 65 | 0 | 90 | 10 | 10.0 | A |
| 66 | 10 | 90 | 0 | 10.0 | A |
| 67 | 0 | 0 | 100 | 7.5 | A |
| 68 | 10 | 0 | 90 | 7.5 | A |
| 69 | 20 | 0 | 80 | 7.5 | B |
| 70 | 30 | 0 | 70 | 7.5 | B |
| 71 | 40 | 0 | 60 | 7.5 | C |
| 72 | 50 | 0 | 50 | 7.5 | B |
| 73 | 60 | 0 | 40 | 7.5 | B |
| 74 | 70 | 0 | 30 | 7.5 | B |
| 75 | 80 | 0 | 20 | 7.5 | A |
| 76 | 90 | 0 | 10 | 7.5 | A |
| 77 | 100 | 0 | 0 | 7.5 | A |
| 78 | 0 | 10 | 90 | 7.5 | B |
| 79 | 10 | 10 | 80 | 7.5 | B |
| 80 | 20 | 10 | 70 | 7.5 | B |
| 81 | 30 | 10 | 60 | 7.5 | C |
| 82 | 40 | 10 | 50 | 7.5 | C |
| 83 | 50 | 10 | 40 | 7.5 | B |
| 84 | 60 | 10 | 30 | 7.5 | A |
| 85 | 70 | 10 | 20 | 7.5 | A |
| 86 | 80 | 10 | 10 | 7.5 | A |
| 87 | 90 | 10 | 0 | 7.5 | A |
| 88 | 0 | 20 | 80 | 7.5 | A |
| 89 | 10 | 20 | 70 | 7.5 | A |
| 90 | 20 | 20 | 60 | 7.5 | A |
| 91 | 30 | 20 | 50 | 7.5 | B |
| 92 | 40 | 20 | 40 | 7.5 | A |
| 93 | 50 | 20 | 30 | 7.5 | A |
| 94 | 60 | 20 | 20 | 7.5 | A |
| 95 | 70 | 20 | 10 | 7.5 | A |
| 96 | 80 | 20 | 0 | 7.5 | A |
| 97 | 0 | 30 | 70 | 7.5 | A |
| 98 | 10 | 30 | 60 | 7.5 | A |
| 99 | 20 | 30 | 50 | 7.5 | A |
| 100 | 30 | 30 | 40 | 7.5 | A |
| 101 | 40 | 30 | 30 | 7.5 | A |
| 102 | 50 | 30 | 20 | 7.5 | A |
| 103 | 60 | 30 | 10 | 7.5 | A |
| 104 | 70 | 30 | 0 | 7.5 | A |
| 105 | 0 | 40 | 60 | 7.5 | A |
| 106 | 10 | 40 | 50 | 7.5 | A |
| 107 | 20 | 40 | 40 | 7.5 | A |
| 108 | 30 | 40 | 30 | 7.5 | A |
| 109 | 40 | 40 | 20 | 7.5 | A |
| 110 | 50 | 40 | 10 | 7.5 | A |
| 111 | 60 | 40 | 0 | 7.5 | A |
| 112 | 0 | 50 | 50 | 7.5 | A |
| 113 | 10 | 50 | 40 | 7.5 | A |
| 114 | 20 | 50 | 30 | 7.5 | A |
| 115 | 30 | 50 | 20 | 7.5 | A |
| 116 | 40 | 50 | 10 | 7.5 | A |
| 117 | 50 | 50 | 0 | 7.5 | A |
| 118 | 0 | 60 | 40 | 7.5 | A |
| 119 | 10 | 60 | 30 | 7.5 | A |
| 120 | 20 | 60 | 20 | 7.5 | A |
| 121 | 30 | 60 | 10 | 7.5 | A |
| 122 | 40 | 60 | 0 | 7.5 | A |
| 123 | 0 | 70 | 30 | 7.5 | A |
| 124 | 10 | 70 | 20 | 7.5 | A |
| 125 | 20 | 70 | 10 | 7.5 | A |
| 126 | 30 | 70 | 0 | 7.5 | A |
| 127 | 0 | 80 | 20 | 7.5 | A |
| 128 | 10 | 80 | 10 | 7.5 | A |
| 129 | 20 | 80 | 0 | 7.5 | A |
| 130 | 0 | 90 | 10 | 7.5 | A |
| 131 | 10 | 90 | 0 | 7.5 | A |
| 132 | 0 | 100 | 0 | 7.5 | A |
| 133 | 0 | 0 | 100 | 5.0 | A |
| 134 | 10 | 0 | 90 | 5.0 | B |
| 135 | 20 | 0 | 80 | 5.0 | B |
| 136 | 30 | 0 | 70 | 5.0 | B |
| 137 | 40 | 0 | 60 | 5.0 | B |
| 138 | 50 | 0 | 50 | 5.0 | C |
| 139 | 60 | 0 | 40 | 5.0 | C |
| 140 | 70 | 0 | 30 | 5.0 | B |
| 141 | 80 | 0 | 20 | 5.0 | B |
| 142 | 90 | 0 | 10 | 5.0 | A |
| 143 | 100 | 0 | 0 | 5.0 | A |
| 144 | 0 | 10 | 90 | 5.0 | B |
| 145 | 10 | 10 | 80 | 5.0 | B |
| 146 | 20 | 10 | 70 | 5.0 | B |
| 147 | 30 | 10 | 60 | 5.0 | B |
| 148 | 40 | 10 | 50 | 5.0 | C |
| 149 | 50 | 10 | 40 | 5.0 | B |
| 150 | 60 | 10 | 30 | 5.0 | B |
| 151 | 70 | 10 | 20 | 5.0 | B |
| 152 | 80 | 10 | 10 | 5.0 | A |
| 153 | 90 | 10 | 0 | 5.0 | A |
| 154 | 0 | 20 | 80 | 5.0 | A |
| 155 | 10 | 20 | 70 | 5.0 | A |
| 156 | 20 | 20 | 60 | 5.0 | A |
| 157 | 30 | 20 | 50 | 5.0 | B |
| 158 | 40 | 20 | 40 | 5.0 | B |
| 159 | 50 | 20 | 30 | 5.0 | A |
| 160 | 60 | 20 | 20 | 5.0 | A |
| 161 | 70 | 20 | 10 | 5.0 | A |
| 162 | 80 | 20 | 0 | 5.0 | A |
| 163 | 0 | 30 | 70 | 5.0 | A |
| 164 | 10 | 30 | 60 | 5.0 | A |
| 165 | 20 | 30 | 50 | 5.0 | A |
| 166 | 30 | 30 | 40 | 5.0 | A |
| 167 | 40 | 30 | 30 | 5.0 | A |
| 168 | 50 | 30 | 20 | 5.0 | A |
| 169 | 60 | 30 | 10 | 5.0 | A |
| 170 | 70 | 30 | 0 | 5.0 | A |
| 171 | 0 | 40 | 60 | 5.0 | A |
| 172 | 10 | 40 | 50 | 5.0 | A |
| 173 | 20 | 40 | 40 | 5.0 | A |
| 174 | 30 | 40 | 30 | 5.0 | A |
| 175 | 40 | 40 | 20 | 5.0 | A |
| 176 | 50 | 40 | 10 | 5.0 | A |
| 177 | 60 | 40 | 0 | 5.0 | A |
| 178 | 0 | 50 | 50 | 5.0 | A |
| 179 | 10 | 50 | 40 | 5.0 | A |
| 180 | 20 | 50 | 30 | 5.0 | A |
| 181 | 30 | 50 | 20 | 5.0 | A |
| 182 | 40 | 50 | 10 | 5.0 | A |
| 183 | 50 | 50 | 0 | 5.0 | A |
| 184 | 0 | 60 | 40 | 5.0 | A |
| 185 | 10 | 60 | 30 | 5.0 | A |
| 186 | 20 | 60 | 20 | 5.0 | A |
| 187 | 30 | 60 | 10 | 5.0 | A |
| 188 | 40 | 60 | 0 | 5.0 | A |
| 189 | 0 | 70 | 30 | 5.0 | A |
| 190 | 10 | 70 | 20 | 5.0 | A |
| 191 | 20 | 70 | 10 | 5.0 | A |
| 192 | 30 | 70 | 0 | 5.0 | A |
| 193 | 0 | 80 | 20 | 5.0 | A |
| 194 | 10 | 80 | 10 | 5.0 | A |
| 195 | 20 | 80 | 0 | 5.0 | A |
| 196 | 0 | 90 | 10 | 5.0 | A |
| 197 | 10 | 90 | 0 | 5.0 | A |
| 198 | 0 | 100 | 0 | 5.0 | A |
| 199 | 0 | 0 | 100 | 2.5 | A |
| 200 | 10 | 0 | 90 | 2.5 | A |
| 201 | 20 | 0 | 80 | 2.5 | B |
| 202 | 30 | 0 | 70 | 2.5 | C |
| 203 | 40 | 0 | 60 | 2.5 | B |
| 204 | 50 | 0 | 50 | 2.5 | B |
| 205 | 60 | 0 | 40 | 2.5 | C |
| 206 | 70 | 0 | 30 | 2.5 | B |
| 207 | 80 | 0 | 20 | 2.5 | B |
| 208 | 90 | 0 | 10 | 2.5 | A |
| 209 | 100 | 0 | 0 | 2.5 | A |
| 210 | 0 | 10 | 90 | 2.5 | B |
| 211 | 10 | 10 | 80 | 2.5 | B |
| 212 | 20 | 10 | 70 | 2.5 | C |
| 213 | 30 | 10 | 60 | 2.5 | C |
| 214 | 40 | 10 | 50 | 2.5 | C |
| 215 | 50 | 10 | 40 | 2.5 | C |
| 216 | 60 | 10 | 30 | 2.5 | C |
| 217 | 70 | 10 | 20 | 2.5 | B |
| 218 | 80 | 10 | 10 | 2.5 | B |
| 219 | 90 | 10 | 0 | 2.5 | A |
| 220 | 0 | 20 | 80 | 2.5 | A |
| 221 | 10 | 20 | 70 | 2.5 | A |
| 222 | 20 | 20 | 60 | 2.5 | B |
| 223 | 30 | 20 | 50 | 2.5 | B |
| 224 | 40 | 20 | 40 | 2.5 | B |
| 225 | 50 | 20 | 30 | 2.5 | B |
| 226 | 60 | 20 | 20 | 2.5 | A |
| 227 | 70 | 20 | 10 | 2.5 | A |
| 228 | 80 | 20 | 0 | 2.5 | A |
| 229 | 0 | 30 | 70 | 2.5 | A |
| 230 | 10 | 30 | 60 | 2.5 | A |
| 231 | 20 | 30 | 50 | 2.5 | A |
| 232 | 30 | 30 | 40 | 2.5 | A |
| 233 | 40 | 30 | 30 | 2.5 | A |
| 234 | 50 | 30 | 20 | 2.5 | A |
| 235 | 60 | 30 | 10 | 2.5 | A |
| 236 | 70 | 30 | 0 | 2.5 | A |
| 237 | 0 | 40 | 60 | 2.5 | A |
| 238 | 10 | 40 | 50 | 2.5 | B |
| 239 | 20 | 40 | 40 | 2.5 | A |
| 240 | 30 | 40 | 30 | 2.5 | A |
| 241 | 40 | 40 | 20 | 2.5 | A |
| 242 | 50 | 40 | 10 | 2.5 | A |
| 243 | 60 | 40 | 0 | 2.5 | A |
| 244 | 0 | 50 | 50 | 2.5 | A |
| 245 | 10 | 50 | 40 | 2.5 | B |
| 246 | 20 | 50 | 30 | 2.5 | A |
| 247 | 30 | 50 | 20 | 2.5 | A |
| 248 | 40 | 50 | 10 | 2.5 | A |
| 249 | 50 | 50 | 0 | 2.5 | A |
| 250 | 0 | 60 | 40 | 2.5 | A |
| 251 | 10 | 60 | 30 | 2.5 | A |
| 252 | 20 | 60 | 20 | 2.5 | A |
| 253 | 30 | 60 | 10 | 2.5 | A |
| 254 | 40 | 60 | 0 | 2.5 | A |
| 255 | 0 | 70 | 30 | 2.5 | A |
| 256 | 10 | 70 | 20 | 2.5 | A |
| 257 | 20 | 70 | 10 | 2.5 | A |
| 258 | 30 | 70 | 0 | 2.5 | A |
| 259 | 0 | 80 | 20 | 2.5 | A |
| 260 | 10 | 80 | 10 | 2.5 | A |
| 261 | 20 | 80 | 0 | 2.5 | A |
| 262 | 0 | 90 | 10 | 2.5 | A |
| 263 | 10 | 90 | 0 | 2.5 | A |
| 264 | 0 | 100 | 0 | 2.5 | A |

**Supplementary Table 3. Testing data points for the SVM classifier.**

| ID (–) | MXene (wt.%) | CNF (wt.%) | Gelatin (wt.%) | Mixture Loading (mg mL^–1^) | Grade (A/B/C) |
| --- | --- | --- | --- | --- | --- |
| 1 | 49 | 11 | 40 | 10.0 | A |
| 2 | 24 | 42 | 34 | 10.0 | C |
| 3 | 65 | 12 | 23 | 10.0 | A |
| 4 | 20 | 16 | 64 | 10.0 | A |
| 5 | 38 | 33 | 29 | 10.0 | C |
| 6 | 30 | 49 | 21 | 10.0 | B |
| 7 | 55 | 35 | 10 | 10.0 | A |
| 8 | 18 | 62 | 21 | 10.0 | C |
| 9 | 31 | 25 | 45 | 10.0 | C |
| 10 | 10 | 47 | 43 | 10.0 | B |
| 11 | 49 | 11 | 40 | 7.5 | B |
| 12 | 24 | 42 | 34 | 7.5 | A |
| 13 | 65 | 12 | 23 | 7.5 | A |
| 14 | 20 | 16 | 64 | 7.5 | A |
| 15 | 38 | 33 | 29 | 7.5 | A |
| 16 | 30 | 49 | 21 | 7.5 | A |
| 17 | 55 | 35 | 10 | 7.5 | A |
| 18 | 18 | 62 | 21 | 7.5 | A |
| 19 | 31 | 25 | 45 | 7.5 | A |
| 20 | 10 | 47 | 43 | 7.5 | A |
| 21 | 49 | 11 | 40 | 5.0 | B |
| 22 | 24 | 42 | 34 | 5.0 | A |
| 23 | 65 | 12 | 23 | 5.0 | B |
| 24 | 20 | 16 | 64 | 5.0 | A |
| 25 | 38 | 33 | 29 | 5.0 | A |
| 26 | 30 | 49 | 21 | 5.0 | A |
| 27 | 55 | 35 | 10 | 5.0 | A |
| 28 | 18 | 62 | 21 | 5.0 | A |
| 29 | 31 | 25 | 45 | 5.0 | A |
| 30 | 10 | 47 | 43 | 5.0 | A |
| 31 | 49 | 11 | 40 | 2.5 | B |
| 32 | 24 | 42 | 34 | 2.5 | A |
| 33 | 65 | 12 | 23 | 2.5 | B |
| 34 | 20 | 16 | 64 | 2.5 | A |
| 35 | 38 | 33 | 29 | 2.5 | A |
| 36 | 30 | 49 | 21 | 2.5 | A |
| 37 | 55 | 35 | 10 | 2.5 | A |
| 38 | 18 | 62 | 21 | 2.5 | A |
| 39 | 31 | 25 | 45 | 2.5 | B |
| 40 | 10 | 47 | 43 | 2.5 | A |

**Supplementary Table 4. Training data points for the prediction model.**

| Loop # (–) | ID  (–) | MXene (wt.%) | CNF (wt.%) | Gelatin (wt.%) | GA (+/–) | Mixture Loading (mg mL^–1^) | $\sigma_{30}$  (kPa) | $R_{0}$  (Ω) |
| --- | --- | --- | --- | --- | --- | --- | --- | --- |
| 1 | 1 | 12.4 | 40.1 | 47.4 | – | 10.0 | 7.8 | 1.0$\times$10^10^ |
| 1 | 2 | 13.1 | 27.0 | 59.9 | – | 10.0 | 8.9 | 1.0$\times$10^10^ |
| 1 | 3 | 33.3 | 35.6 | 31.1 | – | 10.0 | 7.3 | 1.5$\times$10^2^ |
| 1 | 4 | 11.2 | 76.5 | 12.3 | – | 10.0 | 10.3 | 1.0$\times$0^10^ |
| 1 | 5 | 71.0 | 11.5 | 17.4 | – | 10.0 | 7.1 | 3.6$\times$10^0^ |
| 1 | 6 | 60.6 | 27.7 | 11.7 | – | 10.0 | 10.5 | 3.0$\times$10^0^ |
| 1 | 7 | 33.3 | 54.1 | 12.7 | – | 10.0 | 12.1 | 1.0$\times$10^2^ |
| 1 | 8 | 46.9 | 16.2 | 36.9 | – | 10.0 | 9.4 | 4.9$\times$10^0^ |
| 1 | 9 | 20.2 | 67.9 | 11.9 | – | 10.0 | 13.8 | 4.8$\times$10^2^ |
| 1 | 10 | 12.4 | 40.1 | 47.4 | – | 7.5 | 3.3 | 1.0$\times$10^10^ |
| 1 | 11 | 13.1 | 27.0 | 59.9 | – | 7.5 | 3.2 | 1.0$\times$10^10^ |
| 1 | 12 | 33.3 | 35.6 | 31.1 | – | 7.5 | 2.0 | 1.1$\times$10^4^ |
| 1 | 13 | 11.2 | 76.5 | 12.3 | – | 7.5 | 2.9 | 3.0$\times$10^7^ |
| 1 | 14 | 71.0 | 11.5 | 17.4 | – | 7.5 | 4.7 | 8.2$\times$10^0^ |
| 1 | 15 | 33.3 | 54.1 | 12.7 | – | 7.5 | 4.1 | 1.6$\times$10^4^ |
| 1 | 16 | 20.2 | 67.9 | 11.9 | – | 7.5 | 7.8 | 1.0$\times$10^4^ |
| 1 | 17 | 12.4 | 40.1 | 47.4 | – | 5.0 | 0.7 | 1.0$\times$10^10^ |
| 1 | 18 | 13.1 | 27.0 | 59.9 | – | 5.0 | 0.7 | 1.0$\times$10^10^ |
| 1 | 19 | 11.2 | 76.5 | 12.3 | – | 5.0 | 1.0 | 2.2$\times$10^7^ |
| 1 | 20 | 60.6 | 27.7 | 11.7 | – | 5.0 | 0.6 | 9.3$\times$10^2^ |
| 1 | 21 | 33.3 | 54.1 | 12.7 | – | 5.0 | 1.3 | 2.8$\times$10^1^ |
| 1 | 22 | 20.2 | 67.9 | 11.9 | – | 5.0 | 1.9 | 5.5$\times$10^3^ |
| 2 | 23 | 11.2 | 76.5 | 12.3 | – | 2.5 | 0.2 | 1.8$\times$10^7^ |
| 2 | 24 | 60.6 | 27.7 | 11.7 | – | 2.5 | 0.3 | 7.5$\times$10^1^ |
| 2 | 25 | 33.3 | 54.1 | 12.7 | – | 2.5 | 0.2 | 9.3$\times$10^2^ |
| 2 | 26 | 20.2 | 67.9 | 11.9 | – | 2.5 | 0.1 | 1.0$\times$10^6^ |
| 2 | 27 | 60.6 | 27.7 | 11.7 | – | 7.5 | 5.0 | 2.6$\times$10^1^ |
| 2 | 28 | 33.3 | 54.1 | 12.7 | – | 7.5 | 4.6 | n/a |
| 2 | 29 | 20.2 | 67.9 | 11.9 | – | 7.5 | 4.5 | n/a |
| 2 | 30 | 30.4 | 31.9 | 37.7 | + | 10.0 | 7.9 | 1.1$\times$10^2^ |
| 2 | 31 | 61.5 | 25.9 | 12.6 | + | 10.0 | 12.7 | 4.0$\times$10^3^ |
| 2 | 32 | 23.0 | 64.7 | 12.3 | + | 10.0 | 16.1 | 4.3$\times$10^2^ |
| 2 | 33 | 27.5 | 32.2 | 40.3 | + | 7.5 | 5.0 | 1.5$\times$10^2^ |
| 2 | 34 | 26.8 | 62.3 | 10.9 | + | 7.5 | 7.1 | 9.4$\times$10^1^ |
| 2 | 35 | 68.4 | 21.3 | 10.4 | + | 7.5 | 7.1 | 4.9$\times$10^1^ |
| 2 | 36 | 64.1 | 15.4 | 20.5 | – | 10.0 | 8.5 | 1.4$\times$10^0^ |
| 2 | 37 | 10.9 | 18.9 | 70.2 | – | 10.0 | 3.3 | 1.0$\times$10^10^ |
| 2 | 38 | 9.0 | 31.4 | 59.6 | – | 10.0 | 8.8 | 1.0$\times$10^10^ |
| 2 | 39 | 24.8 | 54.1 | 21.1 | – | 10.0 | 10.2 | 3.1$\times$10^5^ |
| 2 | 40 | 9.9 | 67.7 | 22.3 | – | 10.0 | 9.9 | 1.0$\times$10^10^ |
| 2 | 41 | 77.0 | 9.7 | 13.3 | + | 10.0 | 6.2 | 1.7$\times$10^0^ |
| 2 | 42 | 51.7 | 25.8 | 22.5 | + | 10.0 | 6.4 | 5.0$\times$10^0^ |
| 3 | 43 | 21.9 | 28.9 | 49.3 | + | 10.0 | 6.4 | 1.0$\times$10^10^ |
| 3 | 44 | 37.1 | 41.6 | 21.3 | + | 10.0 | 5.6 | 4.6$\times$10^10^ |
| 3 | 45 | 77.6 | 13.1 | 9.3 | – | 7.5 | 3.7 | 1.7$\times$10^0^ |
| 3 | 46 | 21.5 | 25.6 | 53.0 | – | 7.5 | 1.7 | 5.1$\times$10^7^ |
| 3 | 47 | 47.8 | 33.2 | 19.0 | – | 7.5 | 4.4 | 2.2$\times$10^1^ |
| 3 | 48 | 27.4 | 39.6 | 33.0 | + | 7.5 | 4.1 | 6.3$\times$10^6^ |
| 3 | 49 | 19.2 | 45.6 | 35.2 | + | 7.5 | 3.8 | n/a |
| 3 | 50 | 13.0 | 46.0 | 41.0 | + | 7.5 | 4.1 | 1.0$\times$10^10^ |
| 3 | 51 | 12.0 | 54.1 | 34.0 | + | 7.5 | 4.6 | 1.0$\times$10^10^ |
| 3 | 52 | 15.3 | 19.3 | 65.4 | – | 5.0 | 0.3 | 1.0$\times$10^10^ |
| 3 | 53 | 38.7 | 48.9 | 12.4 | – | 5.0 | 1.5 | 1.2$\times$10^2^ |
| 3 | 54 | 13.3 | 50.6 | 36.1 | – | 5.0 | 1.1 | 1.0$\times$10^10^ |
| 3 | 55 | 74.4 | 17.5 | 8.1 | + | 5.0 | 2.3 | n/a |
| 3 | 56 | 47.5 | 32.9 | 19.6 | + | 5.0 | 1.8 | 3.1$\times$10^1^ |
| 3 | 57 | 28.2 | 62.9 | 8.9 | + | 5.0 | 1.8 | 9.5$\times$10^2^ |
| 3 | 58 | 69.9 | 17.9 | 12.2 | – | 2.5 | 0.2 | 7.1$\times$10^1^ |
| 3 | 59 | 40.9 | 38.0 | 21.1 | – | 2.5 | 0.9 | 5.5$\times$10^4^ |
| 3 | 60 | 31.1 | 41.0 | 27.9 | – | 2.5 | 0.6 | 1.0$\times$10^10^ |
| 3 | 61 | 30.3 | 23.9 | 45.8 | – | 10.0 | 12.9 | 7.3$\times$10^6^ |
| 3 | 62 | 17.6 | 37.2 | 45.3 | – | 10.0 | 11.2 | 1.0$\times$10^10^ |
| 4 | 63 | 29.7 | 58.5 | 11.8 | – | 10.0 | 13.1 | 7.1$\times$10^2^ |
| 4 | 64 | 13.3 | 62.4 | 24.4 | – | 10.0 | 12.0 | 1.0$\times$10^10^ |
| 4 | 65 | 23.3 | 48.0 | 28.7 | + | 10.0 | 12.9 | 3.1$\times$10^6^ |
| 4 | 66 | 18.9 | 55.1 | 25.9 | + | 10.0 | 13.0 | 3.9$\times$10^5^ |
| 4 | 67 | 60.6 | 22.3 | 17.2 | – | 7.5 | 5.5 | 1.0$\times$10^1^ |
| 4 | 68 | 46.1 | 41.3 | 12.6 | – | 7.5 | 6.1 | 4.8$\times$10^1^ |
| 4 | 69 | 17.4 | 63.2 | 19.4 | – | 7.5 | 5.5 | 2.3$\times$10^6^ |
| 4 | 70 | 36.6 | 25.2 | 38.2 | + | 7.5 | 4.1 | 1.6$\times$10^3^ |
| 4 | 71 | 8.5 | 31.1 | 60.4 | + | 7.5 | 2.9 | 1.0$\times$10^10^ |
| 4 | 72 | 40.1 | 35.7 | 24.3 | + | 7.5 | 5.2 | 3.5$\times$10^2^ |
| 4 | 73 | 20.9 | 40.1 | 39.1 | + | 7.5 | 5.9 | 1.9$\times$10^6^ |
| 4 | 74 | 8.8 | 33.8 | 57.4 | – | 5.0 | 1.1 | 1.0$\times$10^10^ |
| 4 | 75 | 25.8 | 48.5 | 25.7 | – | 5.0 | 1.5 | 2.7$\times$10^6^ |
| 4 | 76 | 12.4 | 60.3 | 27.2 | – | 5.0 | 1.7 | 1.0$\times$10^10^ |
| 4 | 77 | 18.6 | 26.9 | 54.5 | + | 5.0 | 1.0 | 1.0$\times$10^10^ |
| 4 | 78 | 25.3 | 32.3 | 42.4 | + | 5.0 | 1.1 | 3.5$\times$10^7^ |
| 4 | 79 | 21.7 | 37.0 | 41.2 | + | 5.0 | 1.2 | 1.0$\times$10^10^ |
| 4 | 80 | 20.6 | 55.3 | 24.1 | + | 5.0 | 1.6 | 1.0$\times$10^10^ |
| 4 | 81 | 26.0 | 58.5 | 15.6 | – | 2.5 | 0.2 | 5.5$\times$10^6^ |
| 4 | 82 | 41.9 | 47.4 | 10.7 | + | 2.5 | 0.4 | 1.0$\times$10^10^ |
| 5 | 83 | 17.6 | 72.7 | 9.7 | + | 2.5 | 0.4 | 1.0$\times$10^10^ |
| 5 | 84 | 47.0 | 28.0 | 25.1 | – | 10.0 | 7.2 | 2.6$\times$10^1^ |
| 5 | 85 | 52.5 | 31.4 | 16.2 | – | 10.0 | 7.4 | 6.4$\times$10^0^ |
| 5 | 86 | 57.6 | 31.9 | 10.5 | – | 10.0 | 9.6 | 8.8$\times$10^0^ |
| 5 | 87 | 8.5 | 47.8 | 43.7 | – | 10.0 | 13.7 | 1.0$\times$10^10^ |
| 5 | 88 | 52.9 | 18.5 | 28.7 | + | 10.0 | 6.6 | 1.2$\times$10^2^ |
| 5 | 89 | 22.6 | 19.4 | 58.0 | + | 10.0 | 8.2 | 1.0$\times$10^10^ |
| 5 | 90 | 26.2 | 37.0 | 36.8 | + | 10.0 | 14.2 | 1.3$\times$10^7^ |
| 5 | 91 | 42.2 | 45.3 | 12.5 | + | 10.0 | 12.8 | 1.5$\times$10^2^ |
| 5 | 92 | 64.6 | 11.7 | 23.7 | – | 7.5 | 1.7 | 2.8$\times$10^3^ |
| 5 | 93 | 15.4 | 31.9 | 52.8 | – | 7.5 | 3.3 | 1.0$\times$10^10^ |
| 5 | 94 | 53.7 | 38.5 | 7.9 | – | 7.5 | 4.2 | 1.2$\times$10^1^ |
| 5 | 95 | 28.8 | 45.8 | 25.4 | – | 7.5 | 7.3 | 1.0$\times$10^5^ |
| 5 | 96 | 17.9 | 55.4 | 26.7 | – | 7.5 | 8.9 | 1.0$\times$10^10^ |
| 5 | 97 | 52.1 | 27.8 | 20.1 | + | 7.5 | 4.4 | 6.2$\times$10^1^ |
| 5 | 98 | 20.4 | 34.6 | 45.0 | + | 7.5 | 5.5 | 1.0$\times$10^10^ |
| 5 | 99 | 39.5 | 49.3 | 11.2 | + | 7.5 | 5.9 | 3.1$\times$10^2^ |
| 5 | 100 | 53.3 | 34.5 | 12.2 | – | 5.0 | 1.3 | 3.6$\times$10^1^ |
| 5 | 101 | 22.9 | 42.7 | 34.4 | – | 5.0 | 1.3 | 1.0$\times$10^10^ |
| 5 | 102 | 9.2 | 82.1 | 8.7 | – | 5.0 | 2.2 | 1.0$\times$10^10^ |
| 6 | 103 | 41.4 | 34.9 | 23.7 | + | 5.0 | 1.9 | 1.4$\times$10^4^ |
| 6 | 104 | 16.3 | 45.1 | 38.6 | + | 5.0 | 1.7 | 1.0$\times$10^10^ |
| 6 | 105 | 8.4 | 48.1 | 43.5 | + | 5.0 | 1.6 | n/a |
| 6 | 106 | 43.1 | 42.0 | 14.9 | – | 10.0 | 7.3 | 3.7$\times$10^1^ |
| 6 | 107 | 32.0 | 45.6 | 22.5 | – | 10.0 | 8.7 | 1.9$\times$10^4^ |
| 6 | 108 | 36.7 | 48.6 | 14.7 | – | 10.0 | 9.3 | 1.0$\times$10^2^ |
| 6 | 109 | 34.0 | 50.9 | 15.1 | – | 10.0 | 6.2 | 1.3$\times$10^4^ |
| 6 | 110 | 37.5 | 51.6 | 10.9 | – | 10.0 | 7.0 | 1.0$\times$10^3^ |
| 6 | 111 | 23.4 | 52.5 | 24.0 | + | 10.0 | 9.9 | 1.5$\times$10^6^ |
| 6 | 112 | 17.8 | 23.5 | 58.7 | – | 7.5 | 1.2 | 1.0$\times$10^10^ |
| 6 | 113 | 34.9 | 41.8 | 23.3 | – | 7.5 | 4.0 | 1.4$\times$10^4^ |
| 6 | 114 | 10.0 | 45.2 | 44.7 | – | 7.5 | 5.1 | 1.0$\times$10^10^ |
| 6 | 115 | 24.4 | 60.2 | 15.4 | – | 7.5 | 5.7 | 1.1$\times$10^5^ |
| 6 | 116 | 30.3 | 38.3 | 31.4 | + | 7.5 | 8.1 | 3.7$\times$10^2^ |
| 6 | 117 | 15.0 | 75.0 | 9.9 | + | 7.5 | 5.6 | 2.0$\times$10^5^ |
| 6 | 118 | 20.5 | 31.8 | 47.7 | – | 5.0 | 1.2 | 1.0$\times$10^10^ |
| 6 | 119 | 43.4 | 32.1 | 24.5 | – | 5.0 | 1.5 | 2.0$\times$10^3^ |
| 6 | 120 | 19.5 | 51.8 | 28.8 | – | 5.0 | 2.2 | 1.0$\times$10^10^ |
| 6 | 121 | 45.3 | 30.7 | 24.0 | + | 5.0 | 1.4 | n/a |
| 6 | 122 | 18.5 | 39.0 | 42.6 | + | 5.0 | 2.3 | 1.0$\times$10^10^ |
| 7 | 123 | 9.8 | 62.2 | 28.0 | + | 5.0 | 3.1 | 1.0$\times$10^10^ |
| 7 | 124 | 38.1 | 48.2 | 13.7 | – | 2.5 | 0.9 | 4.4$\times$10^4^ |
| 7 | 125 | 20.5 | 62.6 | 16.9 | + | 2.5 | 0.6 | 1.0$\times$10^7^ |
| 7 | 126 | 30.0 | 38.0 | 32.0 | + | 10.0 | 9.1 | n/a |
| 7 | 127 | 15.0 | 55.0 | 30.0 | + | 10.0 | 4.1 | n/a |
| 7 | 128 | 17.0 | 40.0 | 43.0 | – | 10.0 | 7.5 | n/a |
| 7 | 129 | 32.0 | 50.0 | 18.0 | – | 10.0 | 6.3 | n/a |
| 7 | 130 | 30.0 | 32.0 | 38.0 | + | 7.5 | 3.6 | n/a |
| 7 | 131 | 18.0 | 47.0 | 35.0 | + | 7.5 | 4.2 | n/a |
| 7 | 132 | 19.0 | 35.0 | 46.0 | – | 7.5 | 3.2 | n/a |
| 7 | 133 | 32.0 | 42.0 | 26.0 | – | 7.5 | 3.2 | n/a |
| 7 | 134 | 7.0 | 58.0 | 35.0 | – | 2.5 | 0.3 | n/a |
| 7 | 135 | 6.9 | 27.6 | 65.6 | + | 10.0 | 4.6 | n/a |
| 7 | 136 | 81.1 | 16.5 | 2.4 | + | 10.0 | 11.0 | n/a |
| 7 | 137 | 73.8 | 11.7 | 14.5 | + | 10.0 | 6.4 | n/a |
| 7 | 138 | 23.9 | 23.9 | 52.2 | + | 10.0 | 3.7 | n/a |
| 7 | 139 | 12.5 | 47.4 | 40.1 | + | 10.0 | 7.4 | n/a |
| 7 | 140 | 20.8 | 38.5 | 40.7 | + | 10.0 | 5.6 | n/a |
| 7 | 141 | 2.4 | 23.3 | 74.4 | – | 10.0 | 3.1 | n/a |
| 7 | 142 | 23.0 | 38.1 | 38.9 | – | 10.0 | 6.9 | n/a |
| 8 | 143 | 2.8 | 81.2 | 16.0 | – | 7.5 | 3.2 | n/a |
| 8 | 144 | 43.5 | 51.6 | 4.8 | – | 7.5 | 3.6 | n/a |
| 8 | 145 | 26.5 | 42.7 | 30.8 | – | 7.5 | 2.3 | n/a |
| 8 | 146 | 60.7 | 31.4 | 7.9 | – | 7.5 | 2.8 | n/a |
| 8 | 147 | 23.8 | 70.3 | 5.9 | + | 7.5 | 3.7 | n/a |
| 8 | 148 | 76.2 | 5.7 | 18.1 | + | 7.5 | 1.0 | n/a |
| 8 | 149 | 19.0 | 25.4 | 55.6 | + | 7.5 | 1.4 | n/a |
| 8 | 150 | 25.8 | 53.6 | 20.6 | + | 7.5 | 2.9 | n/a |
| 8 | 151 | 1.2 | 25.2 | 73.6 | – | 5.0 | 0.4 | n/a |
| 8 | 152 | 18.0 | 33.2 | 48.8 | + | 5.0 | 1.6 | n/a |
| 8 | 153 | 20.1 | 30.3 | 49.6 | + | 5.0 | 2.8 | n/a |
| 8 | 154 | 35.5 | 41.4 | 23.1 | – | 5.0 | 0.5 | n/a |
| 8 | 155 | 53.5 | 44.9 | 1.6 | + | 5.0 | 2.3 | n/a |
| 8 | 156 | 42.8 | 52.2 | 5.0 | + | 2.5 | 2.2 | n/a |
| 8 | 157 | 10.4 | 18.1 | 71.5 | – | 2.5 | 0.1 | n/a |
| 8 | 158 | 90.3 | 1.5 | 8.3 | – | 2.5 | 0.0 | n/a |
| 8 | 159 | 22.3 | 53.9 | 23.8 | – | 2.5 | 0.3 | n/a |
| 8 | 160 | 36.6 | 39.6 | 23.8 | – | 2.5 | 0.1 | n/a |
| 8 | 161 | 66.7 | 22.7 | 10.6 | + | 2.5 | 0.2 | n/a |
| 8 | 162 | 64.7 | 29.5 | 5.9 | + | 2.5 | 0.4 | n/a |

**Supplementary Table 5. Testing data points for the prediction model.**

| ID  (–) | MXene (wt.%) | CNF (wt.%) | Gelatin (wt.%) | GA (+/–) | Mixture Loading (mg mL^–1^) | $\sigma_{30}$  (kPa) | $R_{0}$  (Ω) |
| --- | --- | --- | --- | --- | --- | --- | --- |
| 1 | 69.5 | 23.6 | 6.9 | + | 10.0 | 5.9 | n/a |
| 2 | 37.1 | 17.4 | 45.5 | – | 10.0 | 2.7 | 1.2$\times$10^3^ |
| 3 | 73.4 | 12.4 | 14.2 | – | 10.0 | 5.5 | 1.6$\times$10^1^ |
| 4 | 19.2 | 24.2 | 56.6 | + | 10.0 | 2.8 | 1.0$\times$10^10^ |
| 5 | 7.3 | 64.8 | 27.9 | + | 10.0 | 3.9 | 1.1$\times$10^7^ |
| 6 | 31.4 | 53.0 | 15.6 | + | 10.0 | 8.2 | n/a |
| 7 | 23.0 | 35.5 | 41.6 | – | 7.5 | 3.5 | 1.0$\times$10^10^ |
| 8 | 36.3 | 56.0 | 7.6 | – | 7.5 | 3.7 | n/a |
| 9 | 88.8 | 5.1 | 6.1 | + | 7.5 | 1.8 | 2.7$\times$10^1^ |
| 10 | 34.3 | 61.2 | 4.5 | + | 7.5 | 2.9 | 1.1$\times$10^3^ |
| 11 | 73.2 | 20.0 | 6.8 | + | 5.0 | 1.3 | 5.1$\times$10^1^ |
| 12 | 31.8 | 60.1 | 8.1 | – | 5.0 | 1.1 | 1.9$\times$10^4^ |
| 13 | 10.9 | 84.2 | 4.8 | – | 5.0 | 3.2 | n/a |
| 14 | 35.2 | 42.0 | 22.8 | + | 5.0 | 0.7 | 6.9$\times$10^3^ |
| 15 | 17.5 | 28.8 | 53.7 | – | 5.0 | 1.9 | 1.0$\times$10^10^ |
| 16 | 4.7 | 52.0 | 43.2 | + | 5.0 | 2.3 | 1.0$\times$10^10^ |
| 17 | 59.8 | 26.0 | 14.2 | – | 2.5 | 0.2 | 1.1$\times$10^3^ |

**Supplementary Table 6. Training dataset for various prediction models based on different sampling methods.**

| **Sampling Method** | **MXene (wt. %)** | **CNF (wt. %)** | **Gelatin (wt. %)** | **Mixture Loading (mg mL^–1^)** | **Grade** |
| --- | --- | --- | --- | --- | --- |
| Random | 67.2 | 28.5 | 4.3 | 7.5 | B |
|  | 1.0 | 46.7 | 52.4 | 10.0 | B |
|  | 32.3 | 38.8 | 28.9 | 10.0 | B |
|  | 53.4 | 46.6 | 0.0 | 2.5 | A |
|  | 52.9 | 2.1 | 45.0 | 7.5 | D |
|  | 11.1 | 54.6 | 34.3 | 2.5 | C |
|  | 39.9 | 56.3 | 3.8 | 2.5 | A |
|  | 8.6 | 46.5 | 44.9 | 7.5 | A |
|  | 30.8 | 19.2 | 50.0 | 2.5 | C |
|  | 42.3 | 29.6 | 28.1 | 7.5 | A |
|  | 56.8 | 32.1 | 11.1 | 10.0 | A |
|  | 46.3 | 33.7 | 19.9 | 2.5 | A |
|  | 21.0 | 38.5 | 40.4 | 7.5 | A |
|  | 28.6 | 45.7 | 25.7 | 5.0 | A |
|  | 44.9 | 25.5 | 29.6 | 10.0 | A |
| Latin Hypercube | 13.5 | 43.9 | 42.7 | 7.5 | B |
|  | 65.1 | 25.3 | 9.6 | 7.5 | A |
|  | 1.3 | 51.8 | 46.9 | 5.0 | C |
|  | 37.1 | 27.3 | 35.6 | 5.0 | B |
|  | 13.2 | 41.6 | 45.2 | 2.5 | D |
|  | 39.9 | 21.1 | 38.9 | 2.5 | C |
|  | 53.2 | 20.8 | 26.0 | 2.5 | B |
|  | 40.8 | 10.6 | 48.6 | 2.5 | C |
|  | 33.5 | 39.8 | 26.7 | 10.0 | B |
|  | 13.3 | 77.2 | 9.6 | 7.5 | B |
|  | 89.1 | 5.3 | 5.6 | 10.0 | B |
|  | 42.6 | 28.7 | 28.7 | 7.5 | B |
|  | 23.5 | 21.9 | 54.6 | 7.5 | B |
|  | 24.7 | 43.2 | 32.1 | 10.0 | B |
|  | 31.1 | 30.7 | 38.1 | 7.5 | C |
| Active  Learning | 12.4 | 40.1 | 47.4 | 10.0 | A |
|  | 33.3 | 35.6 | 31.1 | 10.0 | A |
|  | 11.2 | 76.5 | 12.3 | 10.0 | A |
|  | 71.0 | 11.5 | 17.4 | 10.0 | A |
|  | 33.3 | 54.1 | 12.7 | 10.0 | A |
|  | 13.1 | 27.0 | 59.9 | 7.5 | A |
|  | 33.3 | 35.6 | 31.1 | 7.5 | A |
|  | 11.2 | 76.5 | 12.3 | 7.5 | A |
|  | 20.2 | 67.9 | 11.9 | 7.5 | A |
|  | 12.4 | 40.1 | 47.4 | 5.0 | A |
|  | 13.1 | 27.0 | 59.9 | 5.0 | A |
|  | 60.6 | 27.7 | 11.7 | 5.0 | A |
|  | 33.3 | 54.1 | 12.7 | 5.0 | A |
|  | 11.2 | 76.5 | 12.3 | 2.5 | A |
|  | 33.3 | 54.1 | 12.7 | 2.5 | A |

**Supplementary Table 7. Comparison between experimental and model-predicted** $\sigma_{30}$**and** $R_{0}$ **values in Fig. 3a.**

| Recipe  Number | MXene (wt.%) | CNF (wt.%) | Gelatin (wt.%) | GA (+/–) | Mixture Loading (mg mL^–1^) | Experimental | | Model-Predicted | |
| --- | --- | --- | --- | --- | --- | --- | --- | --- | --- |
|  |  |  |  |  |  | $\sigma_{30}$  (kPa) | $R_{0}$  (Ω) | $\sigma_{30}$  (kPa) | $R_{0}$  (Ω) |
| 1 | 36.7 | 48.6 | 14.7 | – | 10.0 | 9.3 | 1.0$\times$10^2^ | 8.9 | 1.1$\times$10^2^ |
| 2 | 46.1 | 41.3 | 12.6 | – | 7.5 | 6.1 | 4.8$\times$10^1^ | 6.0 | 4.9$\times$10^1^ |
| 3 | 12.4 | 40.1 | 47.4 | – | 5.0 | 0.7 | 1.0$\times$10^10^ | 0.9 | 1.1$\times$10^10^ |
| 4 | 31.1 | 41.0 | 27.9 | – | 2.5 | 0.6 | 1.0$\times$10^10^ | 0.4 | 1.0$\times$10^10^ |
| 5 | 42.2 | 45.3 | 12.5 | + | 10.0 | 12.8 | 1.5$\times$10^2^ | 12.6 | 1.6$\times$10^2^ |
| 6 | 68.4 | 21.3 | 10.4 | + | 7.5 | 7.1 | 5.0$\times$10^0^ | 7.0 | 4.6$\times$10^0^ |
| 7 | 47.5 | 32.9 | 19.6 | + | 5.0 | 1.8 | 3.1$\times$10^1^ | 1.9 | 3.2$\times$10^1^ |
| 8 | 20.5 | 62.6 | 16.9 | + | 2.5 | 0.6 | 1.$\times$10^7^ | 0.6 | 1.0$\times$10^7^ |

**Supplementary Table 8. Property requirements, model-suggested fabrication parameters, and experimental results in Fig. 3c.**

| Property Requirement | | Model-Suggested Fabrication Parameters | | | | | | Experimental Results | |
| --- | --- | --- | --- | --- | --- | --- | --- | --- | --- |
| Mechanical Property | Electrical Property | Recipe Number | MXene (wt.%) | CNF (wt.%) | Gelatin (wt.%) | GA (+/–) | Mixture Loading (mg mL^–1^) | $\sigma_{30}$  (kPa) | $R_{0}$  (Ω) |
| $\sigma_{30}$ > 10.0 kPa | – | 9 | 22.0 | 66.0 | 12.0 | + | 10.0 | 13.8 $\pm$ 0.7 | n/a |
|  |  | 10 | 58.0 | 32.0 | 10.0 | + | 10.0 | 12.2 $\pm$ 0.3 | n/a |
| $\sigma_{30}$ > 10.0 kPa | $R_{0}$ < 10.0 Ω | 11 | 64.0 | 24.0 | 12.0 | + | 10.0 | 12.6 $\pm$ 0.4 | 8.9 $\pm$ 1.8 |
|  |  | 12 | 66.0 | 24.0 | 10.0 | + | 10.0 | 13.9 $\pm$ 1.2 | 7.2 $\pm$ 0.6 |
|  |  | 13 | 62.0 | 26.0 | 12.0 | + | 10.0 | 12.1 $\pm$ 1.0 | 10.1 $\pm$ 1.0 |
|  |  | 14 | 72.0 | 22.0 | 6.0 | + | 10.0 | 14.9 $\pm$ 0.6 | 5.4 $\pm$ 0.2 |

**Supplementary Table 9. Structural features of conductive aerogels extracted from the SEM images in Fig. 4c.**

| FE Model  Name | Mixture Loading (mg mL^–1^) | Average Pore Width  (µm) | Average Pore Height (µm) | Wall Thickness (µm) | Tuning Parameter ($\beta_{imp}$) | Young’s  Modulus  (MPa) | Poisson’s Ratio  ($\nu$) | Pore Density  (ρ) | Pore Aspect Ratio  ( $\psi$) |
| --- | --- | --- | --- | --- | --- | --- | --- | --- | --- |
| High-Density | 10.0 | 18.2 | 6.4 | 0.3 | 0.3 | 3,440.7 | 0.5 | 5.4 × 10^–2^ | 2.8 |
| Medium-Density | 7.5 | 28.3 | 17.8 | 0.3 | 0.3 | 3,440.7 | 0.5 | 2.9 × 10^–2^ | 1.6 |
| Low-Density | 5.0 | 39.1 | 19.0 | 0.3 | 0.3 | 3,440.7 | 0.5 | 2.5 × 10^–2^ | 2.1 |

**Supplementary Table 10. Compositions and mixture loadings of model-suggested conductive aerogels in Fig 5b.**

| Recipe  Number | MXene (wt.%) | CNF (wt.%) | Gelatin (wt.%) | GA (+/–) | Mixture Loading (mg mL^–1^) | $\sigma_{30}$  (kPa) | $R_{0}$  (Ω) |
| --- | --- | --- | --- | --- | --- | --- | --- |
| 15 | 64.1 | 15.4 | 20.5 | – | 10.0 | 8.5 | 1.4 |
| 16 | 77.6 | 13.1 | 9.3 | – | 7.5 | 4.0 | 1.7 |
| 17 | 60.6 | 27.7 | 11.7 | – | 7.5 | 5.3 | 2.7 |
| 18 | 71.0 | 11.5 | 17.4 | – | 10.0 | 7.1 | 3.6 |
| 19 | 51.6 | 38.4 | 10.0 | – | 10.0 | 7.6 | 4.1 |
| 20 | 68.4 | 21.3 | 10.4 | + | 7.5 | 7.1 | 4.9 |
| 21 | 51.7 | 25.8 | 22.5 | + | 10.0 | 6.4 | 5.0 |
| 22 | 71.0 | 11.5 | 17.4 | – | 7.5 | 4.7 | 8.2 |
| 23 | 60.6 | 22.3 | 17.2 | – | 7.5 | 5.5 | 10.3 |
| 24 | 53.7 | 38.5 | 7.9 | – | 7.5 | 4.2 | 12.4 |
| 25 | 47.8 | 33.2 | 19.0 | – | 7.5 | 4.4 | 21.7 |

**Supplementary Table 11. Comparison of our AI/ML framework with the state-of-the-art works.**

| **Independent Variables**  **(# of DOFs)** | **Optimization/Prediction Targets**  **(# of Property Labels)** | **Multi-Property Optimization** | **Sampling Method** | **Experiments Required for New Design Requests** | **Sample Preparation and Characterization Platforms** | **Data Collection Rate** | **Machine Learning Algorithms** | **Ref.** |
| --- | --- | --- | --- | --- | --- | --- | --- | --- |
| Fuel-to-oxidizer ratio, fuel blend, total concentration, anneal temperature  (4 DOFs) | Determining the optimal fabrication parameters for high-conductivity palladium films at low annealing temperatures  (2 property labels) | Yes | Bayesian optimization  (w/ qEHVI acquisition function) | Possible | Ada: Precision 4-axis laboratory robot (N9)/6-axis collaborative robot (UR5e) | 2 data per hour | Gaussian process regression | 3 |
| Column count, column outer radius, column thickness, twisted angle  (4 DOFs) | Pinpointing the optimal structural parameters for high compression toughness  (1 property label) | No | Bayesian optimization | Possible | BEAR: 3D printers/robotic arm/scale/universal testing machine | 64 data per day | Gaussian process regression | 4 |
| Reagent injection sequence, reaction time length, volume  (>40 DOFs) | Determining the optimal reaction parameters for synthesizing hetero-nanostructures with high $\lambda_{AP}$, $R_{PV}$, $I_{PL}$  (3 property labels) | Yes | Reinforcement learning | Possible | AlphaFlow: A self-driven fluidic lab consisting of reagent injection, droplet oscillation, optical sampling, phase separation, and waste collection. | 2 data per hour | Reinforcement learning | 5 |
| **MXene loading, CNF loading, gelatin loading, GA loading, mixture loading**  **(3 DOFs)** | **Aiming to predict across the full parameter space to produce conductive aerogels with customized mechanical and electrical properties**  **(2 property labels)** | **Yes** | **Active learning (w/ A score acquisition function)** | **No** | **OT-2 robot and UR5e-automated compression tester** | **20 data per 2.5 days** | **Support-vector machine**  **/ artificial neural network**  **/ data**  **augmentation** | **This work** |

**Supplementary Table 12. Comparison of our robotics/ML-integrated workflow with the state-of-the-art works regarding the production of conductive MXene aerogels.**

| **Design Strategy** | **Aerogel Composition** | **Aerogel Density**  **(mg cm^–3^)** | **σ_30_**  **(kPa)** | **Electrical Property**  **(–)** | **Electrical Property Measurement** | **Ref.** |
| --- | --- | --- | --- | --- | --- | --- |
| *Quaternary Aerogel Systems with MXene/CNF* | | | | | | |
| **Robotics/ML-integrated workflow** | **MXene/CNF/gelatin/GA** | **2.0 – 14.0** | **0.05 – 16.1** | **1.4 – 10^10^ Ω** | **Two-electrode testing system** | **This**  **work** |
| *Binary Aerogel Systems with MXene/CNF* | | | | | | |
| Design of experiment | MXene/CNF | 4.0 | 0.5 | 2.8$\times$10^1^ S cm^–1^ | Four-point probe | 6 |
|  |  | 4.0 | 0.8 | 4.0$\times$10^–1^ S cm^–1^ |  |  |
| Design of experiment | MXene/CNF | 50 | 7.0 | 2.6$\times$10^1^ Ω cm | Two-electrode testing system | 7 |
|  |  | 50 | 3.4 | 1.4 $\times$10^1^ Ω cm |  |  |
| *Ternary Aerogel Systems with MXene/CNF* | | | | | | |
| Design of experiment | MXene/CNF/CNT | 7.5 | 0.4 | 2.4$\times$10^3^ S cm^–1^ | Four-point probe | 8 |
| Design of experiment | MXene/CNF/PMDI | 18.0 | 40.0 | 1.1$\times$10^0^ Ω cm | Four-point probe | 9 |
| *Other MXene Aerogel Systems* | | | | | | |
| Design of experiment | MTMS treated bacterial cellulose/MXene | 6.2 | 1.6 | 1.2$\times$10^2^ S cm^–1^ | Four-point probe | 10 |
| Design of experiment | MXene/CNT | 9.1 | 0.5 | 4.5$\times$10^2^ S cm^–1^ | Four-point probe | 11 |
| Design of experiment | MXene/PGPDMS | 9.9 | 0.1 | – | – | 12 |
| Design of experiment | MXene/rGO | 12.2 | 7.5 | 3.6$\times$10^1^ S cm^–1^ | Three-electrode setup | 13 |
| Design of experiment | MXene | 12.6 | 1.0 | 5.4$\times$10^1^ S cm^–1^ | Two-electrode testing system | 14 |
| Design of experiment | MXene/ANF | 25.0 | 17.0 | 1.0$\times$10^4^ Ω cm | Two-electrode testing system | 15 |
| Design of experiment | MXene/CNT/ANF | 42.0 | 2.3 | 2.7$\times$10^0^ Ω cm | Four-point probe | 16 |
|  |  | 42.0 | 0.8 | 2.0$\times$10^–1^ Ω cm | Four-point probe |  |
| Design of experiment | MXene/Waterborne polyurethane (WPU) | – | 3.0 | – | – | 17 |
| *Other Conductive Aerogel Systems* | | | | | | |
| Design of experiment | rGO | 2.3 | 6.0 | 1.6$\times$10^1^ S cm^–1^ | Four-point probe | 18 |
| Design of experiment | rGO | 8.5 | 8.2 | 4.3$\times$10^1^ S cm^–1^ | Four-electrode testing system | 19 |
| Design of experiment | GO/PVA | 10.0 | 5.0 | 3.0$\times$10^0^ S cm^–1^ | Source meter | 20 |
| Design of experiment | GO | 25.0 | 1.0 | 9.7$\times$10^0^ S cm^–1^ | Source meter | 21 |

**Abbreviations**

rGO: Reduced graphene oxide

GO: Graphene oxide

MTMS: Methyltrimethoxysilane

CNT: Carbon nanotube

PGPDMS: Glycidoxypropyldimethoxymethylsilane

PVA: Poly(vinyl alcohol)

PMDI: Poly((phenyl isocyanate)-co-formaldehyde)

ANF: Aramid nanofiber

WPU: Waterborne polyurethane

**Supplementary Table 13. Comparison of our AI/ML and data-driven approach with the state-of-the-art works regarding design insight elucidation.**

| **Insight Elucidation Strategy** | **Building Block(s)** | **Discovery Method(s)** | **Design Insight(s)** | **Ref.** |
| --- | --- | --- | --- | --- |
| *Quaternary Aerogel System with MXene/CNF (Our Work)* | | | | |
| AI/ML and data-driven approach | MXene/CNF/gelatin/GA | Spearman’s analysis, SHapley Additive exPlanations (SHAP) model interpretation | 1. The mixture loading had the most significant impact on the mechanical properties of conductive aerogels. 2. The MXene loading had the most significant impact on the electrical properties of conductive aerogels. | This work |
| *Binary Aerogel System with MXene/CNF* | | | | |
| Design of experiment | MXene/CNF | Experimental observation | 1. The addition of 33 wt.% CNF led to a 350% increase in the compressive moduli of conductive aerogels. 2. The addition of 17 wt.% CNF had a minimal impact on the conductivity of conductive aerogels, while the addition of 50 wt.% CNF significantly decreased the conductivity of these aerogels. | 6 |
| Design of experiment | MXene/CNF | Experimental observation | 1. Increasing the MXene loading decreased the compressive strength of conductive aerogels under 33% strain. 2. Increasing the MXene loading from 0.7 to 14 wt.% decreased the resistance of conductive aerogels from 26.3 to 13.8 Ω. | 7 |
| *Ternary Aerogel System with MXene/CNF* | | | | |
| Design of experiment | MXene/CNF/CNT | Experimental observation | 1. The presence of CNF plays a crucial role in maintaining the structural integrity of aerogels. | 8 |
| Design of experiment | MXene/CNF/PMDI | Experimental observation | 1. Adding 30 wt.% CNFs increased the compressive modulus of MXene/CNF aerogels by 155%. 2. The mechanical strength of MXene/CNF aerogels was improved by chemically crosslinking them with PMDI. | 9 |
| *Other MXene Aerogel Systems* | | | | |
| Design of experiment | MTMS treated bacterial cellulose/MXene | Experimental observation | 1. Aerogels fabricated from a 1:1 ratio of silylated bacterial cellulose to MXene maintained a residual height of >95% and exhibited a compressive strength of 6 kPa under 50% strain. 2. Conversely, aerogels produced from a 1:1 ratio of untreated bacterial cellulose and MXene retained a residual height of approximately 70% and displayed a compressive strength of 5 kPa. | 10 |
| Design of experiment | MXene/CNT | Experimental observation | 1. An aerogel, produced from a 95:5 ratio of MXene to CNT, exhibited a plastic deformation of 4.2% and an electrical conductivity of 450 S m^–1^. 2. Another aerogel, produced from a 60:40 ratio of MXene to CNT, showed a plastic deformation of 2.1% and an electrical conductivity of 100 S m^–1^. | 11 |
| Design of experiment | MXene/PGPDMS | Experimental observation | 1. The integration of PGPDMS into the MXene interlayers produced an ultra-soft aerogel. 2. At aerogel densities of 7.0 and 10 mg cm^−3^, the compressive moduli of the PGPDMS/MXene aerogels were 58 and 140 Pa respectively. | 12 |
| Design of experiment | MXene/rGO | Experimental observation | 1. As the MXene loading increased from 25 to 75 wt.%, the aerogels' maximum strain decreased from 95% to 60%. 2. When the annealing temperature rose from room temperature to 300 °C, both the maximum strain and the conductivity of conductive aerogels increased. | 13 |
| Design of experiment | MXene | Experimental observation | 1. The compressive stress and electrical conductivity of MXene aerogels both increased with the rise in aerogel density. 2. Factors such as sheet alignment, pore size, and overall aerogel microstructure influenced the aerogels’ electrochemical properties. | 14 |
| Design of experiment | MXene/ANF | Experimental observation | 1. As the MXene loading increased from 30, 50, to 70 wt.%, the compressive stress of MXene/ANF aerogels decreased by 8.6%, 25.3%, and 30.1%, respectively. 2. The aerogel’s resistance also displayed a similar trend in relation to the MXene loading. | 15 |
| Design of experiment | MXene/CNT/ANF | Experimental observation | 1. As the CNT loading increased, the mechanical properties of the composite aerogels decreased. 2. On the other hand, a higher CNT loading improved the electrical conductivity of these composite aerogels. | 16 |
| Design of experiment | MXene/Waterborne polyurethane (WPU) | Experimental observation | 1. The composite aerogel, made from functionalized cellulose nanocrystal (f-NCC), MXene, and polyurethane, withstood 100 compression–relaxation cycles, maintaining 76.2% of the initial maximum stress. 2. The wood-like microstructure, interconnected network, and interactions between the f-NCC, MXene, and PU matrix improved the compressibility and elasticity of the composite aerogels. | 17 |

**Supplementary Movie 1.** Automatic pipetting robot (i.e., OT-2 robot) capable of preparing mixed dispersions with various MXene/CNF/gelatin/GA ratios and mixture loadings.

**Supplementary Movie 2.** UR5e robotic arm capable of automating the compression tests of conductive MXene aerogels.

**Supporting References**

1 Hung Anh, L. D. & Pásztory, Z. An Overview of Factors Influencing Thermal Conductivity of Building Insulation Materials. *J. Build. Eng.* **44**, 102604 (2021).

2 Overvelde, J. T. B. & Bertoldi, K. Relating Pore Shape to the Non-Linear Response of Periodic Elastomeric Structures. *J. Mech. Phys. Solids* **64**, 351–366 (2014).

3 MacLeod, B. P. *et al.* A Self-Driving Laboratory Advances the Pareto Front for Material Properties. *Nat.Commun.* **13**, 995 (2022).

4 Gongora, A. E. *et al.* A Bayesian Experimental Autonomous Researcher for Mechanical Design. *Sci. Adv.* **6**, eaaz1708 (2020).

5 Volk, A. A. *et al.* AlphaFlow: Autonomous Discovery and Optimization of Multi-step Chemistry Using A Self-Driven Fluidic Lab Guided by Reinforcement Learning. *Nat.Commun.* **14**, 1403 (2023).

6 Zeng, Z. *et al.* Nanocellulose-MXene Biomimetic Aerogels with Orientation-Tunable Electromagnetic Interference Shielding Performance. *Adv. Sci.* **7**, 2000979 (2020).

7 Xu, W., Wu, Q., Gwon, J. & Choi, J.-W. Ice-Crystal-Templated “Accordion-Like” Cellulose Nanofiber/MXene Composite Aerogels for Sensitive Wearable Pressure Sensors. *ACS Sustainable Chem. Eng.* **11**, 3208–3218 (2023).

8 Xu, T. *et al.* Nanocellulose-Assisted Construction of Multifunctional MXene-Based Aerogels with Engineering Biomimetic Texture for Pressure Sensor and Compressible Electrode. *Nano-Micro Lett.* **15**, 98 (2023).

9 Wu, N. *et al.* Ultrathin Cellulose Nanofiber Assisted Ambient-Pressure-Dried, Ultralight, Mechanically Robust, Multifunctional MXene Aerogels. *Adv. Mater.* **35**, 2207969 (2023).

10 Zhao, D. *et al.* Multifunctional, Superhydrophobic and Highly Elastic MXene/Bacterial Cellulose Hybrid Aerogels Enabled *via* Silylation. *J. Mater. Chem. A* **10**, 24772–24782 (2022).

11 Deng, Z., Tang, P., Wu, X., Zhang, H.-B. & Yu, Z.-Z. Superelastic, Ultralight, and Conductive Ti_3_C_2_T_x_ MXene/Acidified Carbon Nanotube Anisotropic Aerogels for Electromagnetic Interference Shielding. *ACS Appl. Mater. Interfaces* **13**, 20539–20547 (2021).

12 Shi, X. *et al.* Pushing Detectability and Sensitivity for Subtle Force to New Limits with Shrinkable Nanochannel Structured Aerogel. *Nat. Commun.* **13**, 1119 (2022).

13 Jiang, D. *et al.* Superelastic Ti_3_C_2_T_x_ MXene-Based Hybrid Aerogels for Compression-Resilient Devices. *ACS Nano* **15**, 5000–5010 (2021).

14 Tetik, H. *et al.* 3D Printed MXene Aerogels with Truly 3D Macrostructure and Highly Engineered Microstructure for Enhanced Electrical and Electrochemical Performance. *Adv. Mater.* **34**, 2104980 (2022).

15 Wang, L., Zhang, M., Yang, B., Tan, J. & Ding, X. Highly Compressible, Thermally Stable, Light-Weight, and Robust Aramid Nanofibers/Ti_3_AlC_2_ MXene Composite Aerogel for Sensitive Pressure Sensor. *ACS Nano* **14**, 10633–10647 (2020).

16 Yan, Z. *et al.* MXene/CNTs/Aramid Aerogels for Electromagnetic Interference Shielding and Joule Heating. *ACS Appl. Nano Mater.* **6**, 6141–6150 (2023).

17 Cai, C., Wei, Z., Huang, Y. & Fu, Y. Wood-Inspired Superelastic MXene Aerogels with Superior Photothermal Conversion and Durable Superhydrophobicity for Clean-up of Super-Viscous Crude Oil. *Chem. Eng. J.* **421**, 127772 (2021).

18 Moon, I. K., Yoon, S., Chun, K.-Y. & Oh, J. Highly Elastic and Conductive N-Doped Monolithic Graphene Aerogels for Multifunctional Applications. *Adv. Funct. Mater.* **25**, 6976–6984 (2015).

19 Luo, R. *et al.* Super Durable Graphene Aerogel Inspired by Deep-Sea Glass Sponge Skeleton. *Carbon* **191**, 153–163 (2022).

20 Yang, M. *et al.* Biomimetic Architectured Graphene Aerogel with Exceptional Strength and Resilience. *ACS Nano* **11**, 6817–6824 (2017).

21 Pang, K. *et al.* Hydroplastic Foaming of Graphene Aerogels and Artificially Intelligent Tactile Sensors. *Sci. Adv.* **6**, eabd4045 (2020).
