# Supplementary material for: Machine intelligence accelerated design of conductive MXene aerogels with programmable properties
Source: Nat Commun. 2024 Jun 1;15:4685. doi: 10.1038/s41467-024-49011-8 (PMC11144242; doi:10.1038/s41467-024-49011-8)
Supplement: Supplementary file 4 — Description of Additional Supplementary Files [file 41467_2024_49011_MOESM4_ESM.pdf]

Description of Additional Supplementary Files

File Name: Supplementary Movie 1

Description: Automatic pipetting robot (i.e., OT-2 robot) capable of preparing mixed dispersions with various MXene/CNF/gelatin/GA ratios and mixture loadings.

File Name: Supplementary Movie 2

Description: UR5e robotic arm capable of automating the compression tests of conductive MXene aerogels.
